# Malignant epithelia cells-derived spermine induces APOE+ macrophages to suppress tumor immunity in adenocarcinoma of the esophagogastric junction

**DOI:** 10.3389/fmed.2025.1636699

**Published:** 2025-09-02

**Authors:** Yan Zhang, Zhaoyang Liu, Huihui Sun, Zhenxiang Wang, Ye Chen, Zhiqian Hu, Xinxing Li, Kai Xu, Ying Chen, Yanping Xu, Chenfei Wang, Shuchang Xu

**Affiliations:** ^1^Department of Gastroenterology, Tongji Institute of Digestive Disease, Tongji Hospital, School of Medicine, Tongji University, Shanghai, China; ^2^Key Laboratory of Spine and Spinal Cord Injury Repair and Regeneration of Ministry of Education, Department of Orthopedics, Tongji Hospital, School of Life Science and Technology, Tongji University, Shanghai, China; ^3^Frontier Science Center for Stem Cells, School of Life Sciences and Technology, Tongji University, Shanghai, China; ^4^Department of General Surgery, Tongji Hospital, School of Medicine, Tongji University, Shanghai, China; ^5^Tongji Hospital, Shanghai Key Laboratory of Signaling and Disease Research, Frontier Science Center for Stem Cell Research, School of Life Sciences and Technology, Tongji University, Shanghai, China

**Keywords:** adenocarcinoma of the esophagogastric junction, single-cell RNA sequencing, spatial transcriptomics, cancer metabolism, tumor microenvironment

## Abstract

**Background:**

Adenocarcinoma of the esophagogastric junction (AEG) is increasingly recognized as a distinct gastrointestinal tumor type with a poor prognosis. However, the mechanisms driving AEG progression, particularly the interplay between metabolic reprogramming and the immune microenvironment, remain poorly understood.

**Methods:**

We integrated multi-omics to profile the tumor microenvironment and metabolic reprogramming of AEG. Tumor tissues and paired normal adjacent tissues from AEG patients were subjected to single-cell RNA sequencing (*N*=11), spatial transcriptomics (*N*=4), and metabolomics analysis (*N*=26). Molecular experiments and animal models were used for validation.

**Results:**

Our analysis revealed an AEG-specific malignant subtype originating from the esophagogastric junction, characterized by heightened proliferation and poor differentiation. These malignant cells exhibited metabolic reprogramming marked by hyperactivation of the glutamine-arginine-spermine axis with concomitant spermine accumulation. Spermine was found to drive the polarization of tumor-associated macrophages into an APOE^+^ immunosuppressive phenotype, thereby modulating the tumor immune microenvironment. Mechanistically, spermine promoted the phosphorylation of STAT3, thereby enhancing its binding affinity to the APOE promoter region and leading to enhanced transcriptional activation of APOE.

**Conclusion:**

This study identified AEG-like malignant cells as a high-risk subtype, revealed the metabolic-immune crosstalk driven by the spermine-STAT3-APOE axis in AEG progression, and provided potential targets for AEG metabolic intervention and immunotherapy.

## 1 Introduction

Adenocarcinoma of the esophagogastric junction (AEG) is a clinically challenging malignancy with increasing incidence and poor prognosis (1–3). The unique anatomical location at the esophagogastric junction (EGJ) complicates early detection, precise diagnosis, and effective treatment strategies (4, 5). Although AEG is currently classified within clinical guidelines as a subtype of gastric adenocarcinoma (GAC) and esophageal adenocarcinoma (EAC) for management purposes (6, 7), emerging evidence suggests that it possesses distinct molecular characteristics, metastatic behaviors, and responses to therapy, highlighting the necessity for tailored diagnostic and treatment approaches (8, 9). Despite advancements in molecular subtyping (4, 10–13), the mechanisms driving AEG progression, particularly the interplay between tumor heterogeneity, metabolic reprogramming, and the tumor microenvironment (TME), remain inadequately understood.

Recent studies emphasize that metabolic reprogramming not only supports tumor cell proliferation but also actively influences the immunosuppressive TME by modulating immune cell functions (14, 15). Tumor cells preferentially utilize glycolysis or glutaminolysis to sustain rapid growth, while accumulating immunosuppressive metabolites which impair cytotoxic T cells activation and promotes the expansion of regulatory T cells and tumor-associated macrophages (TAMs), facilitating immune evasion (16). Therefore, understanding the metabolic alterations in AEG tumor cells and their interactions with the TME is essential for improving patient outcomes, as these processes may explain the limited effectiveness of immune therapies and reveal new therapeutic targets to disrupt tumor progression and enhance treatment efficacy.

Apolipoprotein E (APOE), a secreted protein, traditionally recognized for its role in lipid metabolism and neurodegenerative diseases (17), is increasingly implicated in immune regulation and cancer progression. Previous studies have shown that APOE can promote the proliferation and migration of tumor cells (18), inhibit the tumor immune microenvironment (19), and be related to resistance to immunotherapy (20, 21). However, the effect exerted by APOE within tumor immune regulation, especially in AEG TME, remains unclear.

This study aimed to unravel these complexities by integrating single-cell RNA sequencing (scRNA-seq), spatial transcriptomics (ST), and metabolomics to map the tumor heterogeneity, metabolic-immune crosstalk, and immune interactions underpinning AEG progression. We focused on identifying EGJ-specific malignant subtypes, their metabolic alterations, and the microenvironmental niches that sustain their aggressiveness. Our findings uncovered a specific AEG-like malignant cells subtype, originating from the EGJ, characterized by poor differentiation and adverse prognosis. These malignant cells engaged in metabolically driven immunosuppression by secreting spermine to promote the polarization of TAMs toward an APOE^+^ phenotype. This work identified the interaction between malignant epithelial cells and TAMs as a key driver of the spermine-mediated immunosuppressive TME, highlighting a distinct intercellular mechanism within the AEG that promotes tumor progression.

## 2 Materials and methods

### 2.1 Sample collection

73 patients diagnosed with AEG who underwent surgery at Shanghai Tongji Hospital were included in this study (6 for scRNA-seq and ST, 13 for LC–MS untargeted metabolomics and 54 for IHC analysis). None of them had received any prior treatment for this disease, including chemotherapy, radiotherapy, targeted therapy, or biological therapy, and did not have any other malignancies. More detailed clinical information, such as age, sex, TNM staging, and degree of differentiation, was summarized in Supplementary Table S1. The Research Ethics Committees of Shanghai Tongji Hospital approved the study (No. 2021-080), and all patients provided written informed consent.

### 2.2 Cell lines and cell culture

The mouse macrophage cell line RAW264.7, mouse forestomach carcinoma cell line MFC, human monocytic leukemia cell line THP-1 and human AEG cell line OE-19 were purchased from National Collection of Authenticated cell cultures. RAW264.7 and MFC cells were cultured in DMEM medium (Meilunbio) containing 10% fetal bovine serum (FBS, Gibco). THP-1 and OE-19 cells were cultured in RPMI 1640 medium (Meilunbio) containing 10% FBS. All cells were grown at 37°C in a 5% CO2 incubator. THP-1 cells were pretreated with 100 ng/mL PMA for 48 h for differentiating into macrophages before further treatments.

### 2.3 Subcutaneous tumor model

All animal experiments were executed in accordance with the ethical obligations approved by the department of laboratory animal science of Tongji University (No. TJAB09624101). MFC cells (5 × 10^6^) were subcutaneously transplanted into the right dorsal flank of male 5 to 6-week-old 615 mice (BiKai Laboratory Animals). Spermine (5 mg/kg) or equal volume of PBS was intraperitoneally administered into these mice once daily for 2 weeks from the day of tumor cell inoculation. Tumor size was measured with a caliper every 2 days, and tumor volume was calculated using the formula: volume (mm3) = 0.5 × width^2^ × length. Mice were sacrificed by cervical dislocation at day 21, and tumors were harvested for flow cytometry and Immunohistochemistry.

### 2.4 Single-cell RNA-seq

#### 2.4.1 Sample processing and library construction

AEG samples were collected post-surgery, cut into ~1 mm^3^ pieces, and suspended in 5 mL digestion buffer (2 mg/mL Collagenase type II, 200 U/mL DNase I). After 45 min incubation at 37°C with shaking, the suspensions were filtered through a 100 μm filter and centrifuged at 400 g, 4°C for 10 min. Erythrocytes were lysed for 2 min using a red blood cell lysis solution. The cell suspensions were passed through a 40 μm filter and centrifuged again. The pellets were re-suspended in PBS with 0.04% BSA, and cell viability was confirmed (>80%) using trypan blue exclusion. The single-cell suspensions were counted with AO-PI (LUNA, D23001) and adjusted to 700–1,200 cells/μL for library preparation using the Chromium Next GEM Single Cell 3′ Kit v3.1 (10x Genomics). Libraries were sequenced on the Illumina NovaSeq 6,000 System (paired-end, 150 bp). Sequencing was performed by Outdo Biotech Co., Ltd. (Shanghai, China).

#### 2.4.2 Data processing

Alignment, filtering, barcode counting, and unique molecular identifier (UMI) counting of the sequencing data were performed using Cell Ranger 7 (10x Genomics, version 6.0.0) with default parameters and mapped to the GRCh38 human reference genome to generate feature-barcode matrix for each sample. Raw feature-barcode matrices were processed using the Seurat R package (version 4.9.9). Cells with mitochondrial RNA rate <= 20% and non-mitochondrial gene counts > 1,000 were kept for the following analysis. Highly variable genes were calculated using “FindVariableFeatures” function in Seurat with “vst” method and nfeatures = 2000. Normalization and variance stabilization were performed using “SCTransform v2” function in Seurat with default parameters. All the data processing steps described on above were performed on each sample, respectively. After quality control and general data processing, the Seurat objects of all samples were integrated using “PrepSCTIntegration” and “merge” functions in Seurat based on the selected integration genes which were identified using “SelectIntegrationFeatures” function. The merged Seurat object was used in the further analysis.

#### 2.4.3 Dimension reduction, batch effect removal and clustering

For dimension reduction, we selected 2000 integration genes as highly variable genes and performed principal component analysis (PCA) with 50 components. The batch effect was removed using Harmony (22) R package based on the PCA space with the dimension set to 50. Then the data were visualized using uniform manifold approximation and projection (UMAP) which using the top 30 dimensions in projection space adjusted by Harmony. For clustering, the shared nearest neighbor (SNN) modularity optimization-based clustering algorithm called Louvain was applied to cluster all single cells (resolution = 1). For T cells, B cells, myeloid cells and epithelial cells, the clustering resolution = 0.5.

#### 2.4.4 Cell type annotation

The major cell types were annotated based on the expression of known markers. *CD3D* for T cells; *CD79A* for B cells; *IGKC* for plasma cells; *CD68* for Mono/Macro cells; *KIT* for mast cells; *COL1A2* for fibroblasts; *PECAM1* for endothelial cells; *EPCAM* for epithelial cells. After annotation the major cell types, we re-clustered each major cell type to further annotated minor cell types. The minor cell types were named by the cell type and the representative marker genes of each cluster. The cluster-specific marker genes were identified using “FindAllMarkers” function in Seurat and selected based on average log fold change and adjusted *p* value. For T cells, we used a public single-cell T cell atlas in gastric cancer (23) as reference and using “TransferData” function in Seurat to get the transferred cell type annotation for each single cell in our data. Then refining annotation results using the known marker genes, each cell cluster can be annotated by the cell type with the max proportion in it. For B cells, the known marker genes for B cell sub lineages were used and the cell type signature scores were calculated using “AddModuleScore_UCell” function in Seurat based those marker genes (*IGHD*, *TCL1A*, *YBX3*, *MS4A1* for FBC; *AICDA*, *GCSAM*, *RGS13*, *IRAG2* for GCB; *CD27*, *IGHD*- for MBC). Then each cell cluster can be annotated by the cell type with the max UCell score in it. For myeloid cells, the annotation procedure was the same as B cells (used marker genes: *S100A8*, *VCAN*, *FCN1* for Macro; *CD163*, *C1QA*, *APOC1* for Mono; *CD1A*, *CD1C*, *CLEC10A* for cDC). For epithelial cells, the malignant cells were firstly identified by the epithelial cells with both high CNV scores and high UCell scores of AEG malignant cell marker genes. Then, the rest of epithelial cell were annotated as normal epithelial cells and applied the same annotation procedure described on above (used marker genes: *MUC5AC*, *TFF1*, *MUC6*, *TFF2* for pit mucous cell; *PGA3*, *LIPF*, *PGA4*, *PGC* for chief cell; *ATP4A*, *ATP4B*, *CBLIF* for parietal cell).

### 2.5 Spatial transcriptome

#### 2.5.1 Sample processing

Paired tumor and adjacent tissues from patient 2 and patient 3 in the scRNA-seq dataset were used for ST. After scRNA-seq tissue removal, samples were embedded in OCT and stored at −80°C. ST libraries were prepared using the Visium Spatial Gene Expression Kit (10x Genomics) per manufacturer’s instructions. Samples were sectioned at 10 μM, placed on capture slides, fixed, stained with H&E, and imaged on a Leica CS2. After permeabilization (18 min), reverse transcription and second strand synthesis were performed. Probe (Large PN-1000364) amplification was done with Cq value determined at ~25% of peak fluorescence. Libraries were sequenced on the NovaSeq 6,000 (Illumina). Sequencing was provided by Outdo Biotech Co., Ltd. (Shanghai, China).

#### 2.5.2 Data processing

The raw data were processed using Space Ranger (10x Genomics, version 2.1.1) with default parameters and mapped to the GRCh38 human reference genome to generate feature-barcode matrix for each sample. Processed data were further normalized using “SCTransform v2” function in Seurat with default parameters.

#### 2.5.3 Spatial cell type annotation

Since the spot diameter in 10X Visium slide is larger than the diameter of a single cell, the gene expression profile in each ST spot is actually a mixture of gene expression profiles of multiple cells with similar or different cell states. Therefore, the key point of spatial cell type annotation is to decompose the cell type composition in each spot. We used STRIDE (24) to perform the spatial cell type deconvolution for each ST slide with our annotated scRNA-seq data as reference. For scRNA-seq data, immune cells from all samples were included while the epithelial cells were sample specific. STRIDE is a topic modeling-based method for accurately decomposing and integrating ST slides. In our previous benchmark work, STRIDE exhibited the overall best performance among other published cell-type deconvolution tools, which ensured the accuracy and robustness of the spatial cell type annotation in our study. Based on the cell type proportions inferred by STRIDE, we annotated each spot by the cell type with the biggest proportion in it.

### 2.6 Estimated copy number variations in epithelial cells

The copy number variations (CNV) were estimated based on expression level from scRNA-seq data using CopyKAT (25) R package. All epithelial cells were treated as the query cells while T cells and B cells were treated as the normal cell references. The CopyKAT uses integrative Bayesian approaches to identify genome-wide aneuploidy at 5 MB resolution in single cells, and cells with high CNV scores are considered as malignant cells.

### 2.7 Public bulk RNA-seq data processing and AEG score

The public bulk RNA-seq dataset of adenocarcinoma of the esophagogastric junction (AEG) used in our study including 83 AEG tumors with paired normal adjacent tissues (NATs) (12). The raw RNA-seq data were filtered, normalized and identified differentially expressed genes (DEGs) using limma (26) R package. The DEGs with the log2 fold change <1.2 were removed then the top 50 and bottom 50 of the rest DEGs were selected as the positive and negative marker gene sets of AEG malignant cells. The AEG score, which was the activation score of AEG marker genes in each epithelial cell was calculated using “AddModuleScore_UCell” function in Seurat with both positive and negative AEG marker gene sets.

### 2.8 Public scRNA-seq data processing, EAC score and GAC score

To investigate the potential origin site of AEG malignant cells, we collected 2 public scRNA-seq datasets of EAC and GAC as the references (27, 28). The processed count data were downloaded and imported using Seurat package, then the similar workflow used in our malignant cell annotation were applied to these public data. Normalization and variance stabilization were performed using “SCTransform v2” function in Seurat with default parameters. The major cell type annotations were acquired from their original publications. CopyKAT was applied to epithelial cells to calculate CNV score with the same parameter setting in our data while T and B cells were used as reference normal cells as well. Next, the malignant cells in each public scRNA-seq data can be identified according to CNV scores. Since the cancer type of AEG is adenocarcinoma, we only included malignant cells which sampled from adenocarcinoma sources in further analysis. Moreover, for GAC scRNA-seq data, only samples from body, antrum and distal were included. The filtering is simply performed according to the sample sheets which provided in publications of those public data. After that, we extracted all malignant cells from public scRNA-seq data and calculated the genome-wide correlations between those reference malignant cells and malignant cell identified in our scRNA-seq data. Only the common genes expressed in all scRNA-seq data were included in the correlation calculation. The correlations, which named as EAC scores and GAC scores in our study, represented the overall similarities between AEG malignant cells and malignant cells from EAC and GAC. AEG malignant cells with high EAC or GAC scores suggesting that these cells were likely started from EAC or GAC tissues.

### 2.9 TCGA cohort and survival analysis

The TCGA cohort consisting 248 samples from TCGA-STAD and TCGA-ESCA projects were built and downloaded from GDC data portal. To make samples in this cohort are similar with AEG samples in our study, we only selected TCGA samples with disease type were “adenomas and adenocarcinomas” and “primary tumor.” Moreover, for TCGA-STAD project, only TCGA samples from cardia, fundus of stomach, gastric antrum and pylorus were included and for TCGA-ESCA project, only TCGA samples from esophagus, thoracic esophagus and lower third of esophagus were included. For the selected 248 samples their TPM matrices and clinical sheets were downloaded, and EAC score, GAC score and AEG score were calculated for each TCGA sample in the same way as what we did in scRNA-seq data. The samples in this cohort were classified into 3 groups, including EAC-like, GAC-like and AEG-like, according to the similarity score between 3 cancer types.

Survival analysis is performed using the “survfit” function in “survival” R package. The optimal cutoff is defined as the point with the most significant split. Finally, the survival curve was plotted using “ggsurvplot” function in “survminer” R package.

### 2.10 Malignant cell differentiation score

The malignant cell differentiation state is highly associated with cell malignancy while less differentiated malignant cells have stronger proliferation and migration abilities. To investigate the differentiation states in different AEG subtypes, we used CytoTRACE (29) R package to predict the differentiation score of malignant cells from scRNA-seq data with the subsampling size = 1,000. The higher differentiation score means the cell was less differentiated. Genes associated with stemness and differentiation were then predicted based on their correlation with differentiation scores.

### 2.11 Gene expression programs of epithelial cells

The gene expression programs (GEPs) of epithelial cells were acquired from a public scRNA-seq study of esophageal squamous-cell carcinoma (27). The GEP is a gene set containing genes with a specific function. Total 4 GEPs were used in our study, including terminal differentiation (Terminal diff), epithelial-mesenchymal transition (EMT), oxidative damage (Oxd) and stress response (Stress). The usage of each GEP was evaluated using “AddModuleScore_UCell” function in Seurat.

### 2.12 Metabolic flux intensity

The cellular metabolic states in malignant cells were predicted by Compass (30) Python package based on scRNA-seq data and flux balance analysis (FBA). Because of the poor scalability of Compass, all the reaction flux intensities were predicted on the cell type level. The purpose of FBA is to find an optimal solution of metabolic reaction flux intensities to make the target system has a balance between intake and output of metabolites. Therefore, the output of Compass is a reaction flux intensity matrix. The reaction flux intensity matrix and reaction-metabolite stoichiometric number matrix were then multiplied to get the flux intensity of metabolites.

### 2.13 Cell–cell interaction

The cell–cell interactions (CCIs) between cell types were predicted using CellChat R package (31) with the default parameters based on scRNA-seq data of each sample. CCIs with *p* value <0.01 were considered as significant.

### 2.14 LC–MS untargeted metabolome

#### 2.14.1 Metabolite extraction

100 mg samples were placed in 2 mL centrifuge tubes with 1,000 μL tissue extract (75% methanol: chloroform, 25% H₂O) and ground twice at 50 Hz for 60s each. Samples were sonicated for 30 min at room temperature, incubated on ice for 30 min, and centrifuged at 12,000 rpm, 4°C for 10 min. The supernatant was concentrated, dried, and redissolved in 200 μL 50% acetonitrile with 4 ppm 2-chloro-l-phenylalanine. The supernatant was filtered through a 0.22 μm membrane and transferred for LC–MS detection.

#### 2.14.2 LC–MS/MS analysis

LC was performed using a Vanquish UHPLC system (Thermo Fisher Scientific). Samples were injected onto an ACQUITY UPLC^®^ HSS T3 column (2.1 × 100 mm, 1.8 μm) at 0.3 mL/min with a 2 μL injection and 40°C column temperature. Metabolites were detected using a Q Exactive Focus mass spectrometer (Thermo Fisher Scientific) with ESI ion source, in Full MS-ddMS2 mode. MS1 scans were performed at 70,000 resolution (m/z 100–1,000), followed by MS/MS with HCD at 30 eV and 17,500 resolutions. Dynamic exclusion was applied to remove unwanted data.

#### 2.14.3 Data processing

The raw data were firstly converted to mzXML format by “MSConvert” function in ProteoWizard package (v3.0.8789) (32) and processed using XCMS R package (v3.12.0) (33) for feature detection, retention time correction and alignment. The batch effect was removed by correcting the data based on QC samples. Metabolites with RSD > 30% in QC samples were filtered and then used for subsequent data analysis.

The metabolites were identified by accuracy mass and MS/MS data which were matched with HMDB (34), MassBank (35), KEGG (36), LMSD (37), mzcloud (38) and the metabolite database built by Panomix Biomedical Tech Co., Ltd. (Shuzhou, China). The molecular weight of metabolites was determined according to the mass-to-charge ratio (m/z) of parent ions in MS data. Molecular formula was predicted by parts per million (ppm) and adduction, and then matched with the database to realize MS identification of metabolites. At the same time, the MS/MS data from quantitative table of MS/MS data, were matched with the fragment ions and other information of each metabolite in the database, so as to realize the MS/MS identification of metabolites.

#### 2.14.4 Pathway enrichment

The significantly up-regulated and down-regulated metabolites in tumor samples were identified using MetaboAnalyst (39) package according to the paired Wilcoxon test. Then those metabolites were mapped back to KEGG metabolic pathways and the differential abundance (DA) score were calculated based on the differential fraction of numbers of up and down-regulated metabolites (40).

### 2.15 Flow cytometry analysis

The subcutaneous tumors were isolated and chopped into 1 mm3 pieces. Cell suspensions were collected after the tumor pieces were digested by collagenase and DNase in 37°C water bath for 30 min. Cells from spleen were isolated by mincing with a 5-mL syringe plunger against a 70 μm cell strainer into a 15 mL falcon tube using Roswell Park Memorial Institute (RPMI) medium. The cells were depleted of erythrocytes by RBC lysis buffer (Beyotime, Cat.: C3702). All samples were acquired with the CytoFLEX LX (Beckman Coulter) and analyzed with FlowJo software. The antibodies used were as follows: PE/Cyanine7 anti-mouse CD45 (BioLegend, #103113), FITC anti-mouse CD3 (BioLegend, #100203), FITC anti-mouse/human CD11b (BioLegend, #101205), PE anti-mouse F4/80 (BioLegend, #111603), APC anti-mouse CD206 (BioLegend, #141707), APC anti-human CD206 (BioLegend, #321109), PE anti-human APOE (BioLegend, #803404), FITC anti-mouse CD45 (BioLegend, #103107), PE/Cyanine7 anti-mouse CD3 (BioLegend, #100219), APC anti-mouse CD4 (BioLegend, #100412), PE anti-mouse CD8 (BioLegend, #100708), PE anti-mouse Foxp3 (BioLegend, #320007), APC anti-mouse TNFα (BioLegend, #506307).

### 2.16 Immunohistochemistry

Paraffin sections of tumor samples from 54 AEG patients were obtained from Shanghai Tongji Hospital. After deparaffinization, rehydration, antigen retrieval, and blocking, the arrays were incubated overnight at 4°C with indicated antibodies. The slides were developed with DAB and counterstained with hematoxylin. The images of stained slides were acquired using software VS2000 (Olympus). The staining intensity of each sample was scored by two pathologists blinded to the clinical data by applying a semiquantitative immunoreactivity score.

For subcutaneous MFC tumors, fixation was conducted in 4% paraformaldehyde (Beyotime) and the follow-up procedures, including image scanning were performed by Servicebio (Shanghai, China). The staining intensity of each image (four random images for one tumor sample) was analyzed using the ImageJ software.

The antibodies used were as follows: APOE (Proteintech, #18254-1-AP, #66830-1-Ig), SMS (Proteintech, #68040-1-lg), ACHE (Proteintech, #17975-1-AP), VNN1 (Proteintech, # 21745-1-AP), F4/80 (Servicebio, #GB12027), CD206 (Servicebio, #GB113497), CD8 (CST, #98941).

### 2.17 siRNA transfection

siRNAs targeting human *SMS*, mouse *sms* and negative control (siNC) were designed and synthesized by Tsingke (Beijing, China). The siRNAs were transfected into MFC cells and OE-19 cells using Lipofectamine 2000 (Thermofisher) according to the manufacturer’s instructions.

### 2.18 Real-time qPCR

Total RNA was extracted using EZ-press RNA Purification Kit (EZBioscience). The complementary DNA was synthesized from purified RNA using 4 × Reverse Transcription Master Mix (EZBioscience) according to the manufacturer’s instructions. qRT-PCR was performed using an Applied Biosystem 7,300 plus Sequence Detection System (Applied Biosystems). The cycle threshold (Ct) values were analyzed using the 2^−ΔΔCt^ method, and the final results were presented as relative fold change. The expression of GAPDH served as internal reference. The sequences of primers used were shown in Supplementary Table S5.

### 2.19 Chromatin immunoprecipitation assay

ChIP assay was examined using the ChIP Assay Kit (P2078; Beyotime). Cells were fixed with 1% formaldehyde for 20 min at room temperature. The chromatin digestion, immunoprecipitation and elution were performed according to the manufacturer’s instructions. The enriched DNA was analyzed by qPCR with SYBR Green Master Mix (Yeasen). The primer sequences for ChIP were shown in Supplementary Table S5.

### 2.20 Western blot

Cells were washed with PBS and lysed in radio immune precipitation assay (RIPA) containing 1% protease inhibitor and 1% phosphatase inhibitor at 4°C for 30 min. The cell lysates were heated with 5x Native Gel Sample Loading Buffer (New Cell Molecular Biotech, WB3002) at 95°C for 10 min and subjected and subjected to SDS-PAGE (10%ExpressCast PAGE, New Cell Molecular Biotech, P2012) analysis. The Western blot was performed according to standard protocol. Antibodies to APOE (Proteintech, #66830-1-Ig), STAT3 (Proteintech, #10253-2-AP), p-STAT3 (HUABIO, #ET1603-40), P65 (CST, #8242S), p-P65 (CST, #3033S), and vinculin (Proteintech, #26520-1-AP) were purchased commercially and used according to the manufacturer’s instructions.

### 2.21 Statistical analyzes

Statistical analysis was performed using GraphPad Prism 9 software. Data of bar graphs represents as fold change or percentage relative to control with standard deviation of three independent experiments. Normally distributed data were analyzed using Student’s *t*-test. Abnormal distributed data were analyzed using non-parametric test. The correlations of IHC staining were analyzed using Pearson’s rank test. Statistical significance was defined as a *p* value of less than 0.05. Levels of significance were indicated as ns, not significant, *: *p* < 0.05, **: *p* < 0.01, ***: *p* < 0.001; ****: *p* < 0.0001.

## 3 Results

### 3.1 Decoding tumor microenvironment characteristics through single-cell and spatial transcriptomic profiling

**Figure 1:**
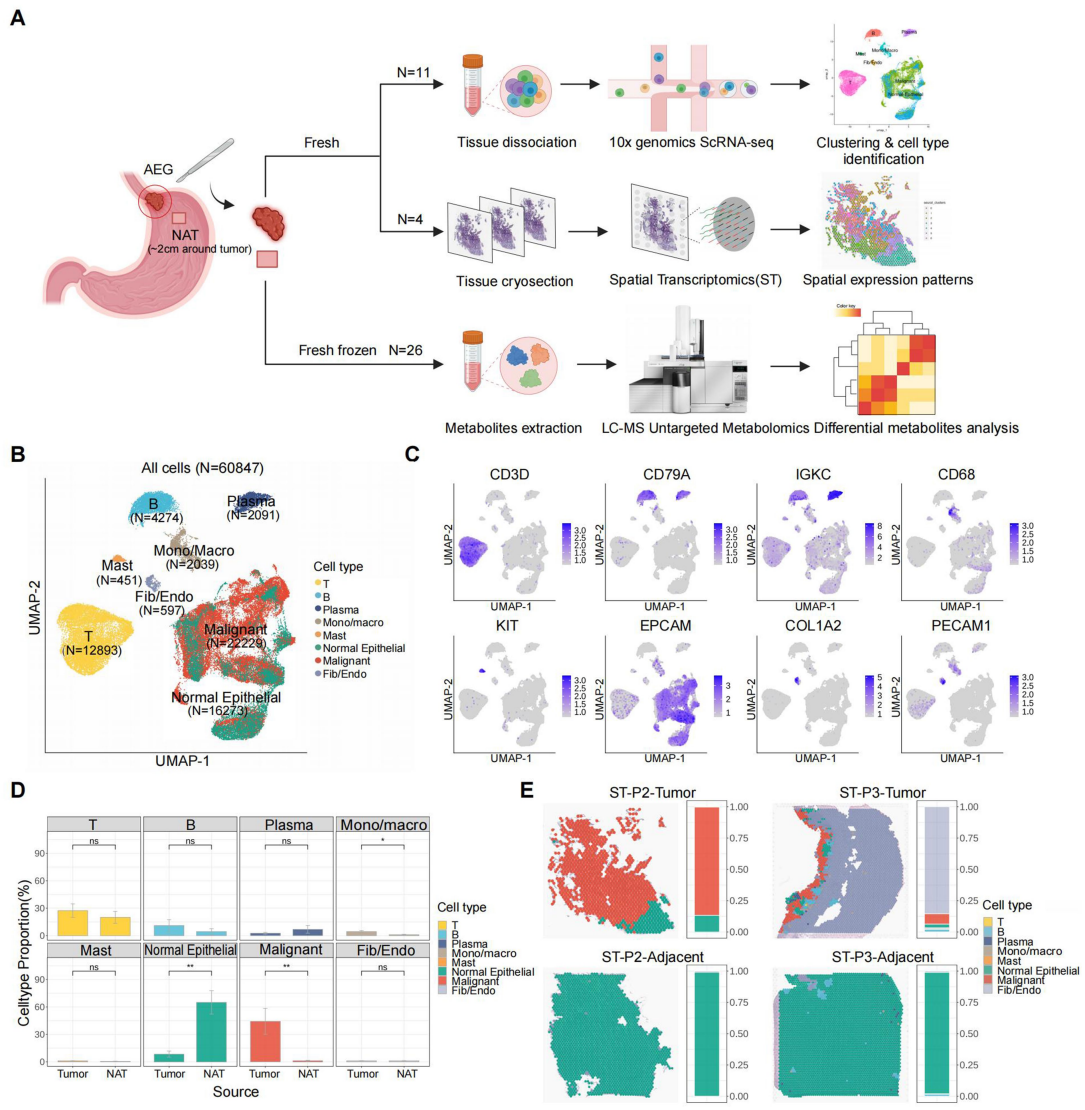
Overview of AEG TME characterized by scRNA-seq and ST. **(A)** Scheme of the study workflow (NAT: normal adjacent tissue). **(B)** UMAP of all the 60,847 cells included in this study, colored by major cell types. **(C)** UMAP of expression levels of the marker gene for each major cell type. **(D)** Major cell type proportion changes between tumor and adjacent tissues (*P*, two-sided Wilcoxon test). *p* values: Mono/Macro (<0.05), normal epithelial (<0.01), malignant (<0.01). **(E)** Spatial major cell type distribution and proportion of each tumor and adjacent ST slide (P2: patient 2, P3: patient 3), colored by major cell types.

To investigate the cell type composition of the AEG microenvironment and explore potential AEG subtypes with distinct molecular signatures, we included 11 samples from 6 patients in our scRNA-seq dataset. Among these samples, 5 patients had paired tumor and NAT samples, while 1 patient had only a tumor sample. Additionally, our ST dataset comprised 4 paired tumor and NAT slides from 2 patients (specifically patient 2 and patient 3 from the scRNA-seq dataset). Furthermore, to gain insights into the metabolic differences between tumor and NAT tissues, we conducted LC–MS untargeted metabolomics analysis on 26 paired samples from 13 AEG patients (Figure 1A; Supplementary Table S1). In the scRNA-seq dataset, we retained a total of 60,847 cells after performing quality control and doublet removal. Following normalization, batch correction, and clustering, we identified 8 major cell types based on established marker genes and AEG malignant scores (Figures 1B,C; Methods). These cell types included T cells (*N* = 12,893, expressing *CD3D*), B cells (*N* = 4,274, expressing *CD79A*), Plasma cells (*N* = 2,091, expressing *IGKC*), Mono/Macro (monocytes or macrophages, *N* = 2,039, expressing *CD68*), Mast (*N* = 451, expressing *KIT*), Normal Epithelial (*N* = 16,273, expressing *EPCAM*), Malignant cells (*N* = 22,229, expressing *EPCAM* and high AEG malignant score) and Fib/Endo (Fibroblasts or Endothelial, *N* = 597, expressing *COL1A2* and *PECAM1* respectively). We did not observe significant proportion changes between tumor and NAT tissues in the scRNA-seq dataset for most immune cell types, except for Mono/Macro cells, which exhibited a higher proportion in the tumor tissue (*p* value < 0.05). As expected, the proportion of normal epithelial cells was significantly decreased in the tumor tissues, while the proportion of malignant cells was significantly elevated (Figure 1D).

To resolve the spatial distribution of cell types, we performed further annotation of the ST slides using the cell types identified in the scRNA-seq analysis (Figure 1E; Supplementary Figure S1A; Methods). Among the 4 slides analyzed, we observed the presence of malignant cells exclusively in 2 tumor tissue slides, while they were absent in the 2 NAT tissue slides. This finding not only confirmed the accuracy of our definition of malignant cells based on the scRNA-seq data but also highlighted the distinct molecular characteristics of the TME. In both tumor tissue slides, a clear demarcation between normal and malignant epithelial cells was evident, representing a canonical boundary structure commonly observed in solid tumor tissues. Notably, in the ST slide obtained from the tumor tissue of patient 3 (ST-P3-Tumor), Fibroblasts or Endothelial cells accounted for over 80% of the spots, and immune cells were clustered around and surrounding the malignant cells. This spatial arrangement suggested the presence of a highly active immune environment and further validated the advantages of leveraging spatial transcriptomics for studying the structure of the TME.

### 3.2 Differential malignant epithelial cellular origins determine tumor heterogeneity in AEG

**Figure 2:**
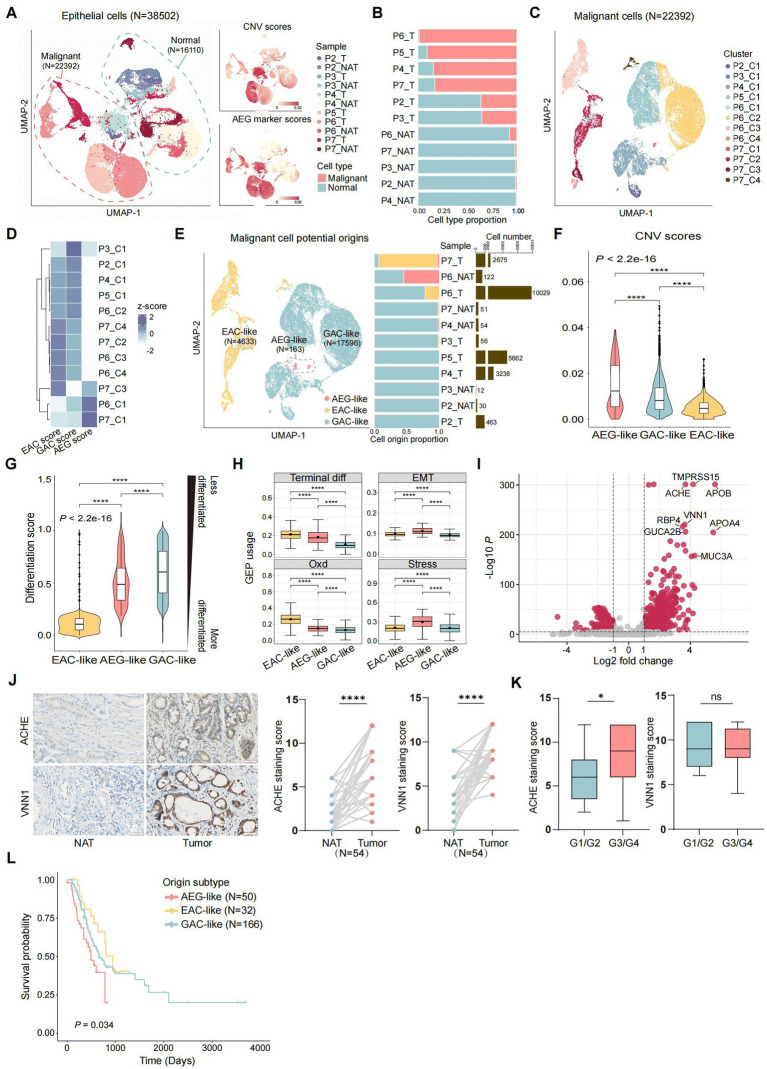
Identification of the potential origins of malignant epithelial cells in AEG. **(A)** UMAP of all the 38,502 epithelial cells from 11 samples (T: tumor, NAT: normal adjacent tissue), colored by the samples, consisted of 22,392 malignant epithelial cells and 16,110 normal epithelial cells (left panel). Malignant and normal epithelial cells were annotated by both CNV scores (top right panel) and AEG marker scores (bottom right panel, markers were acquired from public RNA-seq of AEG). **(B)** Stacked bar plot showing malignant and normal epithelial cell proportions in each sample, ranked by malignant cell proportion (T: tumor, NAT: normal adjacent tissue). **(C)** UMAP of 22,392 malignant epithelial cells, divided into 12 clusters according to the gene expression similarity. **(D)** Heatmap showing the potential origins of each malignant cluster. The origin of each cluster was identified by the whole genome expression similarity scores between each cluster and referenced cells from public EAC, GAC and AEG datasets. **(E)** UMAP of malignant cells, colored by 3 potential origins (left), with the bar plots showed the cell proportion of each malignant subtype (middle) and the cell number of total malignant cells (right) in each sample (ranked by the cell proportion of GAC-like malignant cells). **(F)** Violin plot showing CNV scores of malignant cells, grouped by potential cell origins and ranked by the median CNV score of each group (*P,* two-sided Wilcoxon test, ****: *p* < 0.0001). **(G)** Violin plot showing differentiation scores in each malignant cell subtypes, grouped and colored by malignant cell subtypes. The higher the differentiation score is, the less the cell is differentiated (*P*, two-sided Wilcoxon test, ****: *p* < 0.0001). **(H)** Boxplot showing the usage of identified gene expression program (GEP) in each malignant cell subtype. The black point in each box represents the median value of GEP usages in each malignant cell subtype (Terminal diff: terminal differentiation, EMT: epithelial-mesenchymal transition, Oxd: oxidative damage, Stress: stress response; *P*, two-sided Wilcoxon test, ****: *p* < 0.0001). **(I)** Volcano plot showing differential expression genes between AEG-like malignant cells and other malignant cells. Top-ranked marker genes are labeled (*P*, two-sided Wilcoxon test). **(J)** IHC staining of ACHE and VNN1 in NAT and AEG tumor (*N* = 54; scale bars = 50 μm; *P*, two-sided Wilcoxon test, ****: *p* < 0.0001). **(K)** Correlations of ACHE expression (left) and VNN1 expression (right) with tumor differentiation grade of AEG (G1/G2, *N* = 21; G3/G4, *N* = 33; *P*, Mann–Whitney test, *: *p* < 0.05). **(L)** Kaplan–Meier survival curve compared patients with different malignant origins (*N* = 50 for AEG-like, *N* = 32 for EAC-like, *N* = 166 for GAC-like; *P*, log-rank, global: *p* = 0.034, AEG-like and GAC-like: *p* = 0.043, AEG-like and EAC-like: *p* = 0.0098).

We first investigated the primary source of AEG malignant cells. These cells may originate from the esophagogastric junction, or they may also result from the migration of malignant cells belonging to EAC and GAC. To validate these potential origins, we annotated malignant and normal epithelial cells based on copy number variation (CNV) and gene expression of AEG malignant markers obtained from public bulk RNA-seq data (12). Malignant epithelial cells (*N* = 22,392) were identified as epithelial cells exhibiting high CNV scores and AEG marker scores, while the remaining cells (*N* = 16,110) were classified as normal epithelial cells (Figure 2A; Supplementary Table S2; Methods). Notably, although the majority of malignant cells were found in tumor samples, a small number of malignant cells were also present in adjacent normal tissue samples (Figure 2B). This finding suggests that despite the normal morphology of tissue located 2 cm away from the tumor site, its cellular identity may have already undergone molecular changes. Therefore, molecular identification and classification of AEG could provide more accurate insights. Using known gene markers, we classified normal epithelial cells into 3 major cell types, including chief cells (*N* = 1,557), parietal cells (*N* = 96), and pit mucous cells (PMCs, *N* = 14,457) (Supplementary Figures S2A,B). The composition of these three normal epithelial cell types did not exhibit significant differences between tumor and NAT samples (Supplementary Figures S2C,D).

In contrast to normal epithelial cells, malignant epithelial cells tended to form patient-specific clusters, reflecting the tumor heterogeneity across AEG patients (Figure 2C). To determine the potential origin of each malignant cluster, we extracted the malignant cells from public scRNA-seq datasets of EAC (27) and GAC (28) and treated them as referenced malignant cells (Methods). Subsequently, we calculated the global gene expression correlations to assess the similarities between malignant cells from our AEG dataset and the referenced malignant cells (Figure 2D; Supplementary Figure S2E). Based on their relative similarities with malignant cells from different tumor primary sites, we classified our AEG malignant cells into three groups, indicating the potential origins of malignant cells in AEG (Figure 2E). The EAC-like malignant cells (*N* = 4,633) showed higher similarity scores with EAC malignant cells, suggesting a more probable origin from the esophagus rather than the stomach or EGJ. Similarly, the GAC-like malignant cells (*N* = 17,596) exhibited a higher gastric-derived probability. In contrast, the AEG-like malignant cells (*N* = 163) did not display high similarity with either EAC or GAC cells but showed the highest similarity with the bulk AEG RNA-seq dataset. In this case, we hypothesized that these malignant cells were directly derived from the EGJ. Notably, over half of the samples had a mixed malignant origin, and sample P7_T even exhibited malignant cells with all three origins (refer to Figure 2E), highlighting the complexity of tumor primary sites in AEG.

### 3.3 EGJ-originating AEG-like malignant cells exhibit a poorly differentiated state and associated with adverse clinical outcomes

Although the AEG-like malignant cells were rare in AEG and only present in 3 samples (P7_T, P6_NAT, and P4_NAT), these cells exhibited significantly higher altered CNVs compared to the other 2 subtypes (Figure 2F). Besides, we compared the differentiation states among the 3 malignant subtypes and noted that both AEG-like and GAC-like malignant cells displayed a less differentiated state, indicating a high degree of stemness and proliferation capacity (Figure 2G; Methods). Furthermore, we conducted an analysis of functional gene program enrichments within each malignant subtype (Figure 2H; Methods). Interestingly, the AEG-like malignant cells displayed a higher enrichment of epithelial-mesenchymal transition (EMT) programs, indicating their heightened potential for metastasis. Additionally, these cells demonstrated a pronounced enrichment of stress responses (Stress) and oxidative damage (Oxd) programs. These programs encompass genes that are activated in response to extensive cellular stimuli and oxidative damage (27) (e.g., *EGR1*, *JUN*, and *GXP2*) (Supplementary Table S4).

We further identified marker genes specific to AEG-like malignant cells (Figure 2I; Supplementary Table S3). Among these genes, we validated the expression of ACHE and VNN1 in 54 paired AEG samples using immunohistochemistry (IHC) (Figure 2J). Both ACHE and VNN1 were found to be significantly upregulated in AEG tumor tissues compared with NAT, and ACHE expression was correlated with the grade of tumor differentiation. The expression of ACHE was higher in poorly-differentiated or un-differentiated tumors (G3/G4) than in well-differentiated or moderately-differentiated tumors (G1/G2) (Figure 2K). *ACHE* is an acetylcholine hydrolase that plays a complex role in tumor biology (41). The oncogenic mechanism underlying ACHE in AEG and its potential as a molecular marker for AEG warrant further investigation. Moreover, in the validation cohort collected from the TCGA dataset, patients with high AEG-like malignant signatures showed significantly shorter median survival times than patients with high GAC-like and EAC-like malignant signatures (Figure 2L; Methods).

Collectively, these results suggested that AEG-like malignant cells originating from the EGJ represent an important subtype in AEG, characterized by high malignancy and adverse prognosis.

### 3.4 Metabolic reprogramming drives spermine accumulation in AEG-like malignant cells

**Figure 3:**
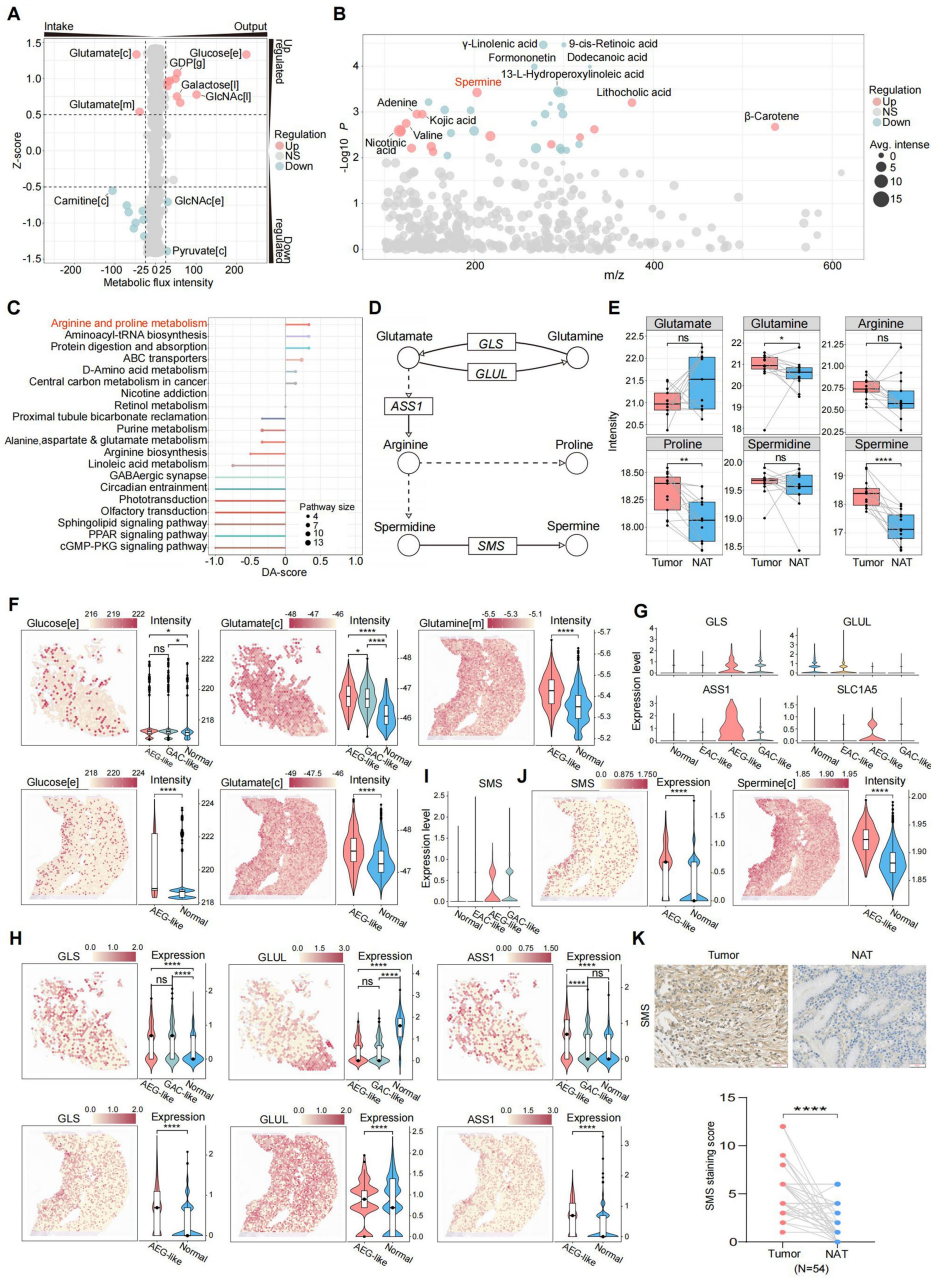
Characterization of metabolic states in AEG-like malignant cells. **(A)** Dot plot showing the intensities of metabolic fluxes in AEG-like malignant cells which were predicted from scRNA-seq data. The metabolic flux intensity reflects the intensity change trend of specific metabolite during the global metabolic exchanges. The positive intensity means the metabolite is accumulated (output), while the negative intensity means consumption (intake). The z-score reflects the variations of metabolic flux intensities in malignant cells, the positive intensity means the change of specific metabolite is up-regulated in AEG-like malignant cell, the positive intensity means down-regulation ([c]: cytoplasm, [e]: extracellular space, [m]: mitochondrion, [l]: lysosome, [g]: Golgi apparatus). **(B)** Dot plot showing the differentially expressed metabolites in AEG tumor samples acquired from LC–MS untargeted metabolomics on 13 paired AEG tumor and normal samples. Each dot represents a metabolite, sized by average intensity in AEG tumor samples and colored by its regulation type (red: up-regulated in AEG tumor samples, blue: down-regulated in AEG tumor samples, gray: no significant difference; *P*, two-sided Wilcoxon test). Top differentially expressed metabolites are labeled. **(C)** Lollipop plot showing the pathway enrichment of differentially expressed metabolites in AEG tumor samples, colored by pathway and sized by the number of matched metabolites in each pathway (DA-score: differential abundance score, positive: pathway is up-regulated in AEG tumor samples, negative: pathway is down-regulated in AEG tumor samples). **(D)** Illustration showing the key metabolites and enzymes in arginine and proline metabolism. The dot line means some reaction steps between linked metabolites are not shown (circle: metabolite, rectangle: enzyme gene, arrow: reaction direction). **(E)** Boxplot showing the intensity changes of key metabolites in arginine and proline metabolism between tumor and normal AEG samples, grouped and colored by samples source (red: tumor, blue: normal; *P,* two-sided Wilcoxon test, ns: *p* > 0.05, *: *p* < 0.05, **: *p* < 0.01, ****: *p* < 0.0001). **(F)** Dim plots showing the metabolic flux distributions on ST slides, which predicted from gene expressions of ST data. Violin plots showing the metabolic flux intensities in spots, grouped by the spot cell types ([c]: cytoplasm, [e]: extracellular space, [m]: mitochondrion; *P,* two-sided Wilcoxon test, ns: *p* > 0.05, *: *p* < 0.05, ****: *p* < 0.0001). **(G)** Violin plots showing the gene expression levels of key enzymes in arginine and proline metabolism in scRNA-seq data, grouped by cell types. **(H)** Spatial plots and violin plots showing the expression levels of key enzyme genes on ST slides in tumor and normal spatial regions (*P*, two-sided Wilcoxon test, ns: *p* > 0.05, ****: *p* < 0.0001). **(I)** Violin plots showing the gene expression levels of *SMS* in scRNA-seq data, grouped by cell types. **(J)** Dim plots and violin plots showing the gene expression levels of *SMS* and intensities of spermine in ST data ([c]: cytoplasm; *P,* two-sided Wilcoxon test, ****: *p* < 0.0001). **(K)** IHC staining of SMS in NAT and AEG tumor (*N* = 54; scale bars = 50 μm; *P*, two-sided Wilcoxon test, ****: *p* < 0.0001).

Cancer is accompanied by profound metabolic disturbances affecting tumor cell proliferation and immune cell differentiation. We first investigated whether specific metabolic states contribute to the invasive phenotype of AEG-like malignant cells. Analyzing metabolite flux intensities inferred from scRNA-seq data, we observed significant alterations in metabolic pathways in AEG-like malignant cells. Notably, these cells exhibited heightened intake of glutamate in both the cytoplasm and mitochondria, while simultaneously accumulating glucose in the extracellular space (Figure 3A; Methods). The high glucose microenvironment can promote the proliferation and suppress apoptosis of malignant cells (42), providing support to the high proliferation ability of AEG-like malignant cells.

To further characterize the metabolic features of AEG-like malignant cells, we performed LC–MS untargeted metabolomics on 26 paired AEG samples (Supplementary Figure S3A; Methods). Our findings revealed significant upregulation of spermine, lithocholic acid, and adenine in AEG tumor samples compared to normal samples. Conversely, several lipids and lipid-like metabolites, including γ-linolenic acid, 9-cis-retinoic acid, and dodecanoic acid, were significantly down-regulated (Figure 3B). Integration of these differential metabolites into metabolic pathways highlighted the arginine and proline metabolism pathways as the most prominently upregulated in tumor samples (Figures 3C,D; Methods). According to our metabolomics data, the intensity of glutamate was decreased in AEG tumor samples, consistent with the predicted up-regulation of glutamate intake in AEG-like malignant cells (Figures 3A,E). Meanwhile, glutamine, proline, and spermine displayed significantly increased intensities in tumor samples (Figure 3E), suggesting metabolic reprogramming characterized by dysregulation of glutamate-arginine-spermine metabolism in AEG-like malignant cells.

We performed metabolic enrichment analysis on ST data to validate and further explore the metabolic reprogramming observed in AEG-like malignant cells. The spots corresponding to AEG-like malignant cells displayed significantly higher glucose intensity compared to spots corresponding to normal cell types, indicating a glucose-rich microenvironment within AEG-like malignant cells (Figure 3F). Additionally, the intake potential of glutamate was higher in AEG-like malignant spots compared to GAC-like malignant spots and other normal spots, consistent with our previous findings. Glutamine also displayed higher intensity in AEG-like malignant spots. Furthermore, gene expression analysis revealed significant upregulation of glutaminase (*GLS*), arginosuccinate synthase 1 (*ASS1*), and glutamine transporter SLC1A5 in AEG-like malignant cells, while the expression of glutamate-ammonia ligase (*GLUL*) was suppressed (Figures 3G,H). These gene expression changes suggested that AEG-like malignant cells exhibit increased uptake of glutamine from the extracellular matrix through glutamine transporters such as *SLC1A5* and glutamine further metabolized into glutamate and arginine under the action of *GLS* and *ASS1*. Arginine was rapidly consumed and converted into spermine under the up-regulation of spermine synthase (*SMS*), leading to spermine accumulation, as confirmed by both scRNA-seq and ST data (Figures 3I,J). Additionally, IHC analysis revealed that the protein levels of SMS were significantly higher in tumor tissues compared to NATs (Figure 3K).

Collectively, these findings indicated that AEG-like malignant cells exhibit a metabolic reprogramming characterized by glucose enrichment, elevated glutamate intake, and enhanced spermine production, providing a solid metabolic foundation for tumorigenesis and tumor proliferation in these cells.

### 3.5 AEG-like malignant cells foster an immunosuppressive TME enriched with Macro_APOE

**Figure 4:**
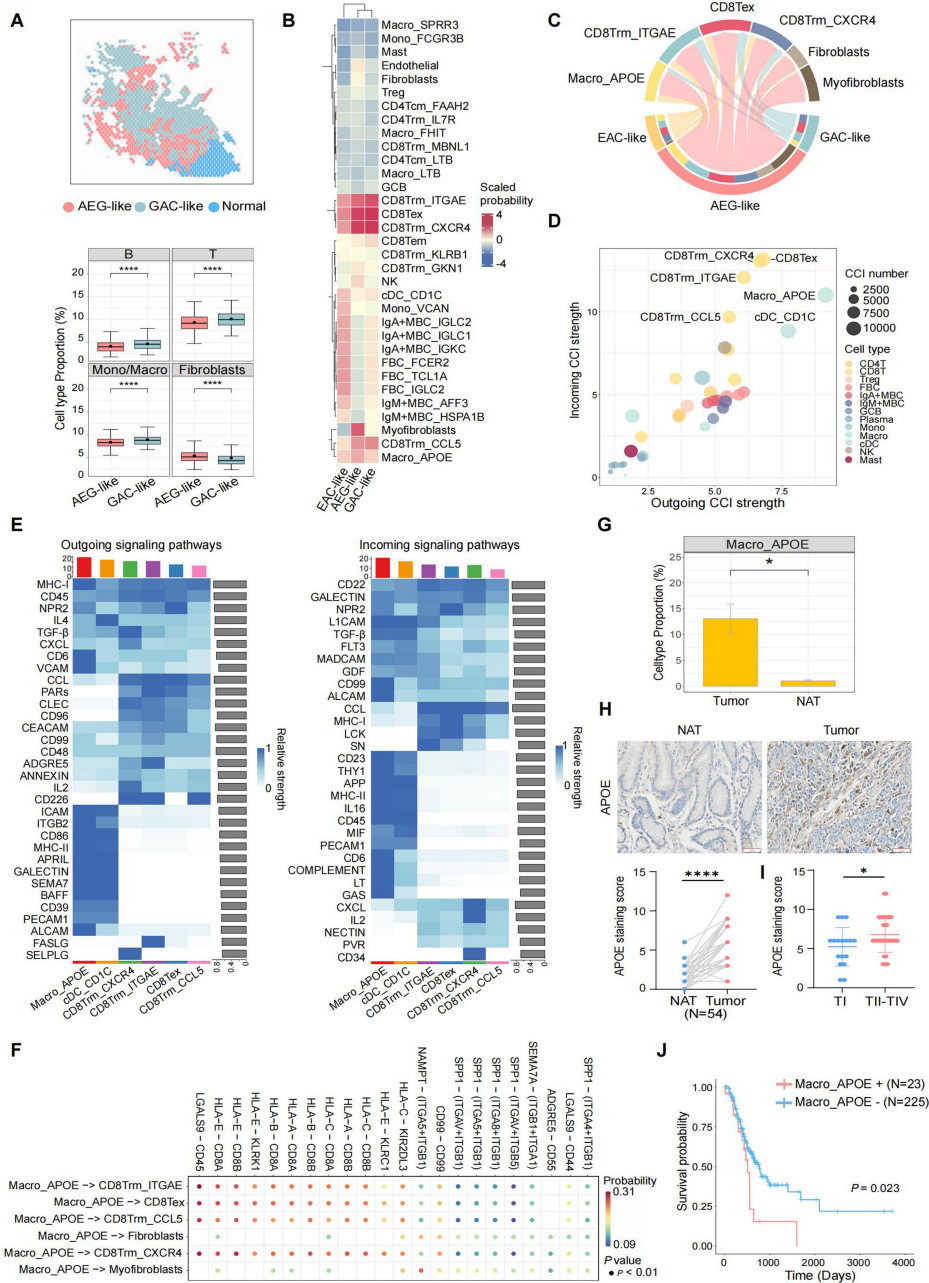
Characterization of AEG immune microenvironment. **(A)** Spatial plot showing the cell type annotation of ST spots (left). The immune and fibroblasts cell type fractions in AEG-like and GAC-like spots are shown in boxplots on the right (*P*, two-sided Wilcoxon test, ****: *p* < 0.0001). **(B)** Clustered heatmap showing the average interaction probability between 3 malignant cell types and other immune and stromal cell types (scaled by cell types). The average interaction probability is indicated by color. **(C)** Chord plot showing the CCIs from 3 malignant cell types to selected target cell types, colored by cell types. The arrow widths are proportional to the interaction strength between connected cell types. The inner bar colors represent the targets that receive signal from the corresponding outer bar. The inner and outer bar size is proportional to the signal strength received by the targets or send by the senders. **(D)** Dot plot showing average incoming and outgoing CCI strengths of each minor cell type, colored by major cell types. The dot size is proportional to the number of total CCIs in each minor cell type. **(E)** Heatmap showing the outgoing (left) and incoming (right) signaling patterns of target cell types in the pathway level. The relative interaction strength is indicated by color. The bar plots showing the total signaling strength of each cell type (top) and the total signaling strength of each signaling pathway (right). **(F)** Dot plot showing the top significant CCIs between Macro_APOE cells and other target cell types. The interaction probability is indicated by color. **(G)** Boxplot showing the cell type proportions of Macro_APOE cells in AEG tumor and NAT (*P*, two-sided Wilcoxon test, *: *p* < 0.05). **(H)** IHC staining of APOE in NAT and AEG tumor (*N* = 54; scale bars = 50 μm; *P*, two-sided Wilcoxon test, ****: *p* < 0.0001). **(I)** Correlations of APOE expression with TNM stages of AEG tumor (TI, *N* = 17; TII-TIV, *N* = 37; *P*, Mann–Whitney test, *: *p* < 0.05). **(J)** Kaplan–Meier survival curve compared patients with different Macro_APOE signature levels (*N* = 23 for Macro_APOE signature high, *N* = 225 for Macro_APOE signature low; *P*, log-rank).

Next, we assessed the impact of AEG-like malignant cells on the TME of AEG. ST data showed significantly lower proportions of immune cells and higher proportions of fibroblasts in AEG-like malignant spots compared to GAC-like malignant spots, indicating the cold and immunosuppressive TME surrounding AEG-like malignant cells (Figure 4A). Then we detected the cell–cell interactions (CCIs) between AEG-like malignant cells with peripheral immune and stromal cell subtypes, established based on a public reference and cluster-specific marker genes (Supplementary Figures S4A–F; Methods). The results revealed that AEG-like malignant cells, as well as GAC-like and EAC-like malignant cells, demonstrated a preference for interacting with APOE^+^ macrophages (Macro_APOE) and specific CD8T cell subtypes, including CD8Trm_ITGAE, CD8Trm_CXCR4, CD8Trm_CCL5, and CD8Tex (Figure 4B). Although all malignant subtypes interacted with Macro_APOE cells and CD8T cells, AEG-like malignant cells showed the strongest CCI strength (Figure 4C), suggesting their heightened potential for immune regulation within the AEG TME.

We then investigated the CCIs between major immune cell subtypes in TME (Methods), and found that certain subtypes of CD8T cells (CD8Tex, CD8Trm_CXCR4, CD8Trm_ITGAE, and CD8Trm_CCL5) and myeloid cells (Macro_APOE and cDC_CD1C) exhibited high CCI activity in both incoming and outgoing signals (Figure 4D). Notably, Macro_APOE cells showed the strongest outgoing CCI strength, indicating their significant role as signal senders in the AEG TME. Outgoing signaling in Macro_APOE cells was enriched in pathways associated with immune response, T cell activation, and inflammatory regulation, such as MHC-I, CD6, and VCAM (Figure 4E). Furthermore, we identified several immunosuppressive CCIs through which Macro_APOE cells modulate T cell activation, such as HLA-E-CD8A/B and LGALS9-CD44/CD45, leading to the establishment of a T cell-suppressive TME (Figure 4F). Additionally, Macro_APOE proportion significantly increased in tumor samples (Figure 4G), which was consistent with the IHC analysis that APOE was highly expressed in AEG tumor tissue compared to NAT in the validate cohort (Figure 4H). Given that APOE serves as a macrophage-specific marker (19, 43), its expression levels directly reflect Macro_APOE abundance. Furthermore, APOE expression was also positively correlated with tumor TNM stages (Figure 4I), and patients with high Macro_APOE cell signatures in the TCGA AEG cohort showed a poor prognosis (Figure 4J). These results suggested that Macro_APOE was enriched in the TME and played pivotal role in the immune suppression and tumor progression of AEG.

### 3.6 Spermine activates the STAT3/APOE axis in macrophage *in vitro*

**Figure 5:**
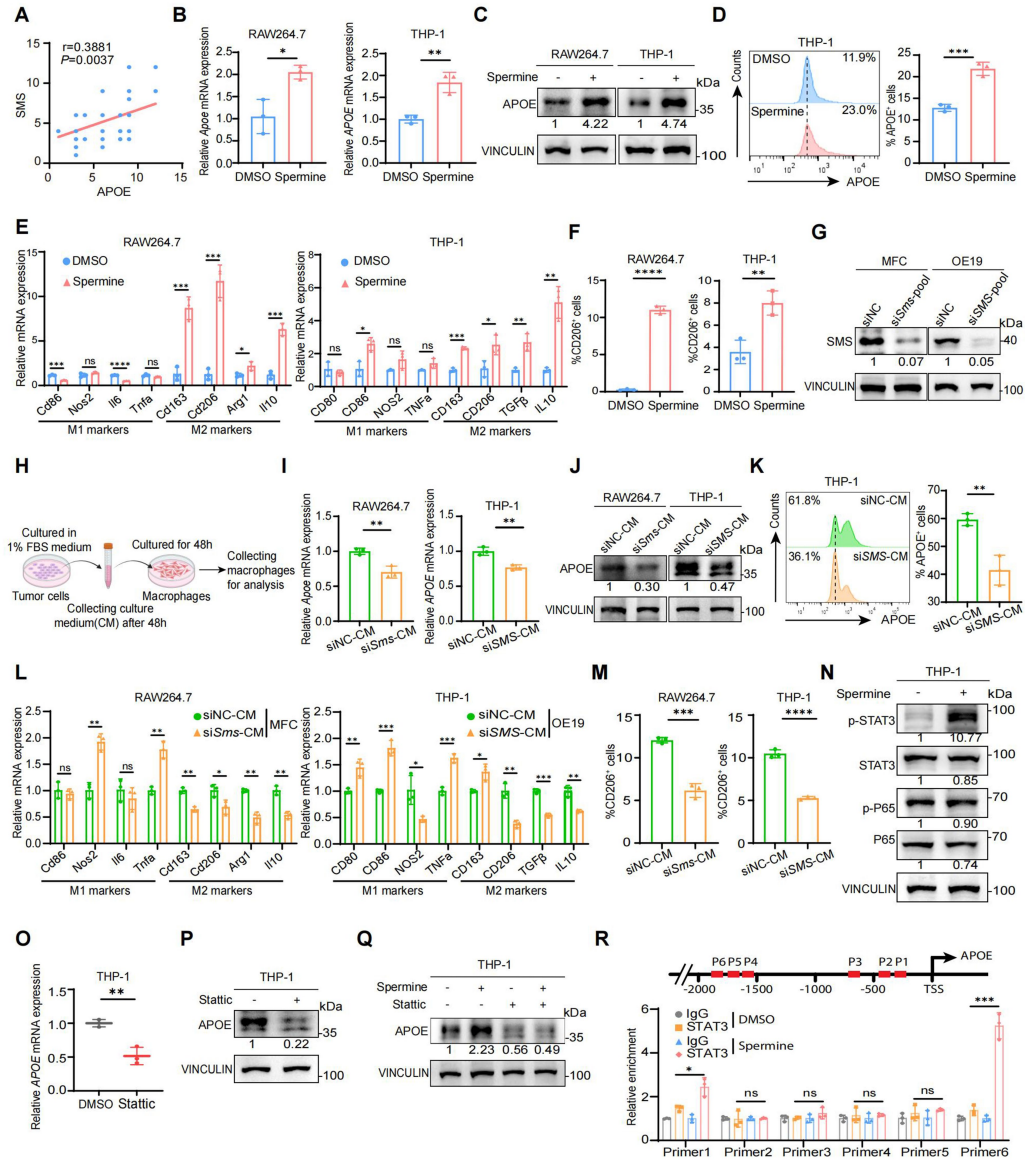
Spermine activates the STAT3/APOE axis in macrophage *in vitro*. **(A)** The correlation analysis of SMS and APOE expressions in human AEG samples (*N* = 54; *P*, Pearson correlation test). **(B)** Real-time qPCR analysis of *APOE* mRNA expression in RAW264.7 and THP-1 treated with DMSO or spermine (10 μM) for 48 h (*N* = 3; *P*, two-tailed unpaired *t*-test, *: *p* < 0.05, **: *p* < 0.01). **(C)** Western blot analysis of APOE protein expression in RAW264.7 and THP-1 treated with DMSO or spermine (10 μM) for 48 h. **(D)** Flow cytometry analysis of APOE^+^ cell proportion in THP-1 treated with DMSO or spermine (10 μM) for 48 h (*N* = 3; *P*, two-tailed unpaired *t*-test, ***: *p* < 0.001). **(E)** Real-time qPCR analysis of M1 markers and M2 markers mRNA expression in RAW264.7 and THP-1 treated with DMSO or spermine (10 μM) for 48 h (*N* = 3; *P*, two-tailed unpaired *t*-test, ns: *p* > 0.05, *: *p* < 0.05, **: *p* < 0.01, ***: *p* < 0.001; ****: *p* < 0.0001). **(F)** Flow cytometry analysis of CD206^+^ cell proportion in RAW264.7 and THP-1 treated with DMSO or spermine (10 μM) for 48 h (*N* = 3; *P*, two-tailed unpaired *t*-test, ***: *p* < 0.001). **(G)** Assessment of the efficiency of SMS knockdown by small interfering RNA in MFC and OE-19 via Western blot. **(H)** Scheme for the process of culture medium (CM) of tumor cells extraction and macrophages cultivation. **(I)** Real-time qPCR analysis of *APOE* mRNA expression in RAW264.7 and THP-1 treated with CM of control or si*SMS* for 48 h (*N* = 3; *P*, two-tailed unpaired *t*-test, *: *p* < 0.05). **(J)** Western blot analysis of APOE protein expression in RAW264.7 and THP-1 treated with CM of control or si*SMS* for 48 h. **(K)** Flow cytometry analysis of APOE^+^ cell proportion in THP-1 treated with CM of control or si*SMS* for 48 h (*N* = 3; *P*, two-tailed unpaired *t*-test, ns: *p* > 0.05, *: *p* < 0.05, **: *p* < 0.01, ***: *p* < 0.001). **(L)** Real-time qPCR analysis of M1 markers and M2 markers mRNA expression in RAW264.7 and THP-1 treated with CM of control or si*SMS* for 48 h (*N* = 3; *P*, two-tailed unpaired *t*-test, **: *p* < 0.01). **(M)** Flow cytometry analysis of CD206^+^ cell proportion in RAW264.7 and THP-1 treated with CM of control or si*SMS* for 48 h (*N* = 3; *P*, two-tailed unpaired *t*-test, **: *p* < 0.01). **(N)** Western blot analysis of STAT3 and P65 total and phosphorylated protein expression in THP-1 treated with DMSO or spermine (10 μM) for 48 h. **(O)** Real-time qPCR analysis of *APOE* mRNA expression in THP-1 treated with DMSO or stattic (10 μM) for 24 h (*N* = 3; *P*, two-tailed unpaired *t*-test, **: *p* < 0.01). **(P)** Western blot analysis of APOE in THP-1 treated with DMSO or stattic (10 μM) for 24 h. **(Q)** Western blot analysis of APOE in THP-1 treated with spermine in the presence or absence of stattic. **(R)** ChIP-qPCR analysis of STAT3 binding to the *APOE* promoter in THP1 treated with DMSO or spermine (10 μM) for 48 h (*N* = 3; *P*, two-tailed unpaired *t*-test, ns: *p* > 0.05, *: *p* < 0.05, ***: *p* < 0.001).

Metabolic changes in tumor cells can modulate immune cell survival and function, ultimately promoting immune suppression and driving tumor progression (44). Due to AEG-like malignant cells showed the strongest CCI strength with Macro_APOE and spermine accumulated in AEG-like malignant cells, further investigation focused on the relationship between spermine and Macro_APOE cells. We analyzed the correlation between SMS and APOE expression in AEG samples by IHC. Notably, the expression of SMS was positively correlated with APOE (Figure 5A), which indicated that spermine was probably positively correlated with Macro_APOE cells. Based on these observations, we speculated that spermine derived from AEG-like malignant cells modulates the immunosuppressive TME in AEG by regulating Macro_APOE cells.

To validate this hypothesis, we first investigated the effect of spermine on macrophage polarization. Macrophages can be generally classified into two subpopulations: proinflammatory M1 phenotype and immunosuppressive M2 phenotype. We detected the expression of APOE, as well as M1 and M2 marker genes, in macrophages treated with spermine. The results demonstrated that spermine significantly elevated the expression of APOE both at mRNA and protein levels (Figures 5B,C). Flow cytometry analysis further confirmed spermine treatment increased Macro_APOE proportion (Figure 5D). Besides, we also observed the increased expression of M2 marker genes, while the expression of M1 marker genes either decreased or remained unaltered (Figure 5E). Similarly, flow cytometry analysis showed spermine treatment increased CD206^+^ macrophages proportion (Figure 5F; Supplementary Figures S5A,B). These phenomena suggested that spermine promoted macrophages polarization toward Macro_APOE, an immunosuppressive M2 phenotype. We then generated *SMS* low-expression tumor cells by small interfering RNA (siRNA) and cultured macrophages in a mixture of tumor cells culture medium (CM) and freshly complete medium (1:1) *in vitro* (Figures 5G,H). We found that macrophages exposed to CM from SMS low-expressing tumor cells were less polarized toward Macro_APOE, and the M2 marker genes expression and the proportion CD206^+^ macrophages also declined (Figures 5I–M; Supplementary Figures S5C,D). These results showed that both exogenously added spermine and tumor cells-derived spermine could polarize macrophages toward the Macro_APOE phenotype.

The NF-κB and STAT3 signaling pathways have been identified as the two primary molecular cascades that regulate M2 polarization in tumor-associated macrophages (45, 46). We therefore investigated whether these two pathways were altered after spermine treatment on macrophages. It is notable that spermine stimulation resulted in a marked intensification of STAT3 phosphorylation, while the phosphorylation levels of P65 remained unaltered (Figure 5N). This finding suggested that spermine activates the STAT3 signaling pathway in macrophages. STAT3 is a well-known transcription factor, phosphorylated STAT3 forms a homodimer, which is then translocated to the nucleus to facilitate transcriptional regulation of target genes (47). Given that spermine regulated APOE at the transcriptional level, we wondered whether STAT3 is a transcription factor for APOE. STAT3 phosphorylation inhibitor (stattic) treatment suppressed APOE expression at both the mRNA and protein level (Figures 5O,P). And the inhibitions of STAT3 signaling efficiently overruled the effect of spermine on APOE (Figure 5Q). ChIP-qPCR further confirmed that STAT3 bound to the APOE promoter, and spermine promoted this binding (Figure 5R).

Taken together, these results indicated that spermine activated the STAT3/APOE axis in macrophages, thereby promoting the polarization of macrophages toward Macro_APOE.

### 3.7 Validation of spermine-mediated immunosuppression through promoting Macro_APOE polarization *in vivo*

**Figure 6:**
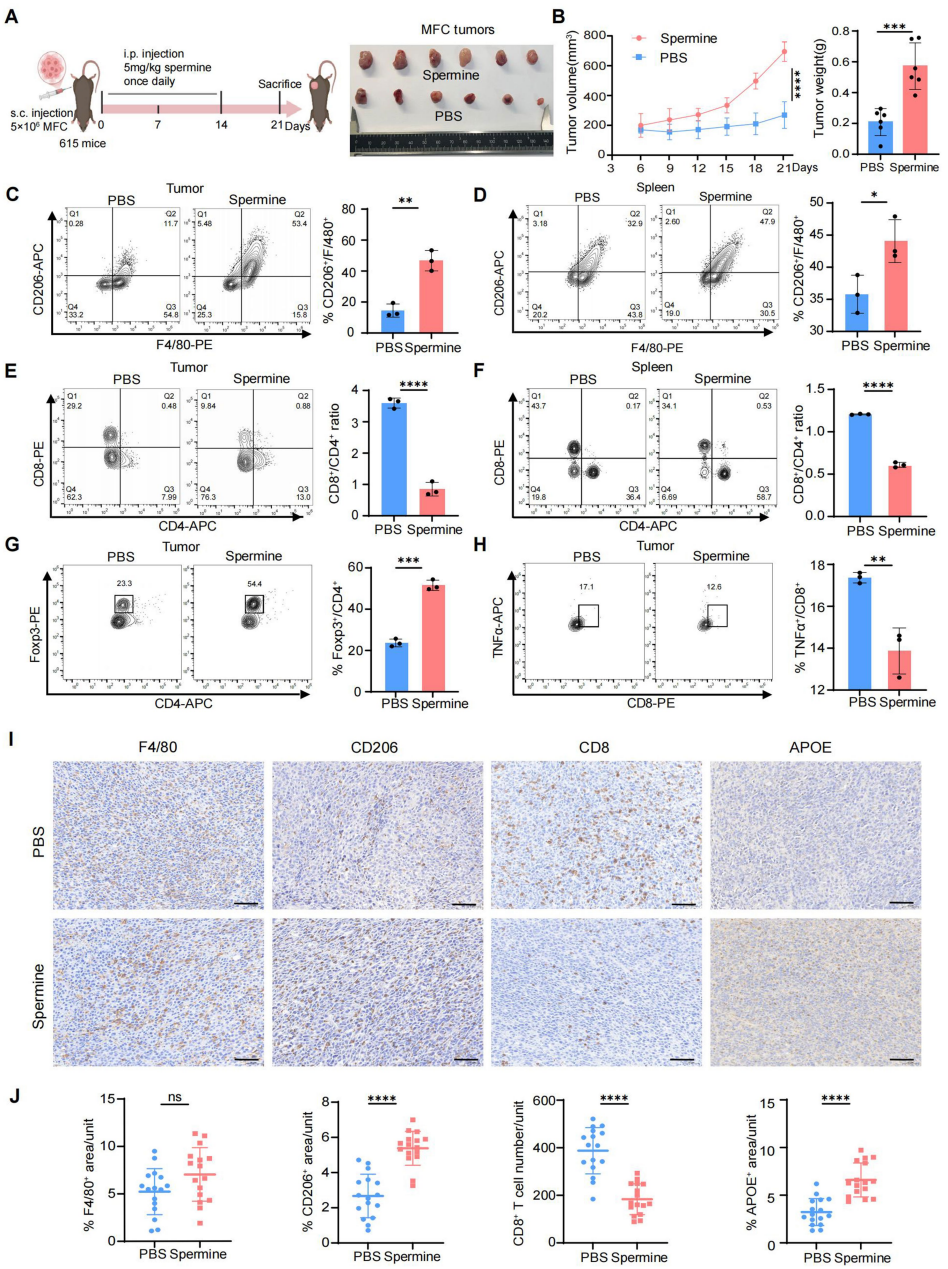
Validation of spermine-mediated immunosuppression through promoting Macro_APOE polarization *in vivo*. **(A)** Scheme for MFC subcutaneous grafts and treatment with spermine or PBS in 615 mice (left). Representative images of the tumors at sacrifice (right) (*N* = 6 for each group). **(B)** Tumor volume (left) and tumor weight (right) in mice in **(A)** (*N* = 6; *P*, two-way ANOVA for left, two-tailed unpaired *t*-test for right, ***: *p* < 0.001; ****: *p* < 0.0001). **(C,D)** Percentage of CD206^+^ macrophages in tumor **(C)** and spleen **(D)** were assessed by flow cytometry (*N* = 3; *P*, two-tailed unpaired *t*-test, *: *p* < 0.05, **: *p* < 0.01). **(E,F)** Percentage of CD8^+^ T and CD4^+^ T cells in tumor (M) and spleen (N) were assessed by flow cytometry (*N* = 3; *P*, two-tailed unpaired *t*-test, ****: *p* < 0.0001). **(G)** Percentage of Treg cells in tumor were assessed by flow cytometry (*N* = 3; *P*, two-tailed unpaired *t*-test, ***: *p* < 0.001). **(H)** Percentage of TNFα^+^CD8^+^ T cells in tumor were assessed by flow cytometry (*N* = 3; *P*, two-tailed unpaired *t*-test, **: *p* < 0.01). **(I)** Representative image of IHC staining of F4/80, CD206, CD8 and APOE in tumor (scale bars = 50 μm). **(J)** Statistical analysis of F4/80^+^, CD206^+^, CD8^+^ and APOE^+^ cell infiltration levels in tumor (*N* = 4, *P*, two-tailed unpaired *t*-test, ns: *p* > 0.05; ****: *p* < 0.0001).

Next, we further investigated the immunomodulatory effects of spermine on the TME *in vivo*. MFC cells were subcutaneously inoculated into the right dorsal flank of 615 mice. Starting from the day of tumor implantation, mice received daily intraperitoneal injections of either spermine (5 mg/kg) or PBS (vehicle control) for 14 consecutive days (48) (Figure 6A). Spermine treatment significantly increased tumor burden, as evidenced by both enlarged tumor volume and elevated tumor weight compared to PBS-treated controls (Figure 6B). Flow cytometric analysis revealed spermine-induced remodeling of immune cell populations. Specifically, we observed increased population of M2 macrophages (CD206^+^F4/80^+^) in tumor tissue and spleen (Figures 6C,D), accompanied by a reduced CD8^+^/CD4^+^ T cell ratio (Figures 6E,F). Tumors from spermine-treated mice exhibited expanded Treg cells (Foxp3^+^CD4^+^) populations and diminished functional cytotoxic T cells (TNFα^+^CD8^+^) (Figures 6G,H). We then examined the level of APOE expression, F4/80^+^ macrophages, CD206^+^ macrophages and CD8^+^T cells using IHC staining (Figure 6I). The results confirmed that spermine treatment promoted macrophages polarization toward Macro_APOE and reduced the CD8^+^T cells infiltration. Notably, although spermine increased the proportion of CD206^+^ macrophages, the total F4/80^+^ macrophage population exhibited no significant alteration, suggesting specific modulation of macrophage polarization rather than global macrophage recruitment (Figure 6J).

Overall, these results demonstrated that spermine induce immunosuppressive TME through promoting Macro_APOE polarization, and inhibiting cytotoxic CD8^+^T infiltration, ultimately facilitating tumor progression.

## 4 Discussion

This study integrates multi-omics and functional analysis to unravel the complex interplay between tumor heterogeneity, metabolic reprogramming, and immunosuppression TME in AEG. A rare yet aggressive subpopulation of AEG-like malignant cells, originating directly from the EGJ, emerged as a critical driver of tumor progression. The AEG-like malignant cells-derived spermine drives macrophage polarization toward Macro_APOE through the activation of STAT3 signaling, thus leading to the establishment of an immunosuppressive TME, ultimately facilitating AEG tumor progression.

The EGJ exposes to both gastric acid and bile reflux, creating a pro-inflammatory environment for tumor cell. Specifically, AEG-like malignant cells demonstrated a pronounced enrichment of stress responses and oxidative damage programs (Figure 2H), indicating that these cells are subject to greater exposure to cellular stimuli and oxidative damage. Besides, the expression of *SMS* in AEG-like malignant cells was higher than that in EAC-like malignant cells and GAC-like malignant cells (Figure 3I), and spatial transcriptomics data further confirmed that spermine accumulated around AEG-like malignant cells (Figure 3J), indicating that tumor metabolism in this area differed from that in the EAC and GAC. These factors may drive AEG-like malignant cell emergence and high malignancy.

Spermine, a key polyamine species, has been widely implicated in oncogenesis. Elevated spermine levels are frequently observed across multiple cancer types (49–52) and correlate with pro-tumorigenic processes including enhanced cellular proliferation, suppressed apoptosis, and transcriptional activation of metastasis-associated genes (49, 50, 53). Beyond its direct oncogenic effects, spermine exerts immunomodulatory functions that contribute to immunosuppressive microenvironments. For instance, spermine could inhibit IFN-I response through suppressing JAK signaling to attenuate autoimmune pathogenesis in systemic lupus erythematosus (54). It also restricts lung inflammation by impairing dendritic cell-mediated cytokine secretion, collectively demonstrating its potent immunosuppressive properties. Additionally, it has also been proved that spermine could regulate the polarization of macrophages. In acute liver injury models, it drives macrophage polarization toward M2 phenotypes via ATG5-dependent autophagy (55). Besides, spermine suppresses innate immune response by significantly reducing the levels of inducible *NOS2* in macrophages responding to *H. pylori* infection (56). In this study, we also found that spermine plays an immunosuppressive role in the TME of AEG. Spermine facilitated the polarization of macrophages toward the Macro_APOE, and enhanced the infiltration of Tregs while simultaneously reducing the infiltration of functional T cells within the tumors. Notably, while we identified that spermine increases APOE transcription via STAT3 phosphorylation in macrophages, the upstream mechanisms driving STAT3 activation and spermine’s broader immunomodulatory effects on other immune cells remain to be further elucidated.

The functional polarization of TAMs plays a pivotal role in shaping immunosuppressive tumor microenvironments (57). Our findings highlight a specific subset of Macro_APOE as key mediators of spermine-driven immunosuppression in AEG. Emerging evidences underscored immunosuppressive crosstalk mediated by Macro_APOE with both tumor cells or immune cells. In pancreatic cancer, Macro_APOE activated CXCL1 and CXCL5 expression through LDL receptor and NF-κB signaling in tumor cells, thereby establishing immunosuppressive niches (19). Similarly, Macro_APOE impaired immunotherapy efficacy via interactions with CD8Tex in triple-negative breast cancer, an effect reversible through APOE blockade (21). Through CCIs network analysis, we confirmed extensive communication hubs mediated by Macro_APOE involving tumor cells and other immune cells, such as CD8Tex populations. However, the precise molecular mechanisms underlying these interactions require systematic interrogation. Macro_APOE has also been shown to directly regulate tumor cell proliferation and invasion. In particular, APOE was identified as a highly specific and effective protein in M2 macrophage-derived exosomes. Exosomes derived from M2 macrophages mediate intercellular transfer of PI3K-Akt signaling pathway activated by APOE in recipient gastric cancer cells, thereby reshaping the migration of cytoskeleton (18). The present study focuses on the immunoregulatory role of APOE. Further investigation is required to determine whether it exerts a direct regulatory effect on tumor cells and, if so, whether this regulatory effect is consistent with the mechanism observed in gastric cancer.

As spermine-STAT3-APOE axis is a key mechanism driving immunosuppression in AEG, targeting any part of the axis may disrupt the metabolic-immune crosstalk and re-sensitize tumors to immunotherapy. Polyamine blockade therapy (PBT) simultaneously targets both endogenous synthesis and exogenous uptake to achieve comprehensive polyamine depletion in the TME. Representative agents include the ornithine decarboxylase (ODC) inhibitor DFMO and the polyamine transport inhibitor AMXT1501. This combinatorial strategy has demonstrated marked synergistic antitumor efficacy in preclinical models (58). In neuroblastoma models, its efficacy has been shown to surpass that of DFMO monotherapy (59). A recent study proved that both DFMO and AMXT-1501 had individual efficacy on tumor regression in hepatocellular carcinoma, and the combination with PD1 inhibitor seemed to result in much better tumor control. Besides, a spermine-restricted diet also be helpful to sensitize the response to immunotherapy (60). Combined inhibition of STAT3 and immune checkpoint blockade (ICB) has shown encouraging results, whereby the addition of STAT3 inhibitors can enhance therapeutic efficacy, and reduce resistance to ICB immunotherapy in parallel. Dasatinib, an indirect STAT3 inhibitor, significantly facilitated anti-CTLA-4 immunotherapy in head and neck squamous cell carcinoma (61), while the combined blockade of IL-6 and PD-L1 remarkably inhibited the growth of pancreatic ductal adenocarcinoma and hepatocellular carcinoma (62, 63). In mice colon cancer model, a combination therapy involving stattic was found to significantly enhance the antitumor T cell response, improve long-term survival, and reduce the immunosuppressive TME, compared to survivin mRNA monotherapy (64). Stattic also showed effective inhibition of the spermine-STAT3-APOE axis in our study, suggesting that STAT3 is a potential target for combination immunotherapy in AEG. APOE blockade has also been reported to enhance the efficacy of ICB therapy. COG 133 TFA is a fragment of APOE peptide, exerting the effect of APOE blockade by competing with APOE holoproteins for binding to low-density lipoprotein (LDL) receptors (65). The combination of COG 133 TFA and anti-PD1 dual treatment can produce significant tumor suppression (21, 66) and COG 133 TFA monotherapy also can reduce the proliferation, invasion and migration of cancer cells (66). Based on the research above, precision targeting of the spermine-STAT3-APOE axis represents a promising strategy to remodel the tumor microenvironment and enhance the efficacy of ICB therapy in AEG.

However, this study has some limitations. First, the scRNA-seq and ST data used to analysis contained only six AEG patients, resulting in a certain bias. These findings need to be further validated in larger cohorts. Second, while adjacent normal tissues serve as a common control in cancer studies, we recognize that molecular alterations due to field cancerization effects or tumor microenvironment influence may exist. This could potentially affect comparative analyzes with tumor tissues. Procurement of healthy donor-matched tissues (e.g., esophagogastric junction) is ethically and logistically challenging for this disease cohort. In future studies, we will incorporate age-matched healthy controls from organ donation programs where feasible.

Collectively, this work established a framework in which metabolic reprogramming and immune suppression converge to fuel AEG progression, offering novel therapeutic targets for this understudied malignancy.

## Data Availability

The datasets presented in this study can be found in online repositories. The names of the repository/repositories and accession number(s) can be found in the article/Supplementary material.

## Supplementary material

SUPPLEMENTARY TABLE S1 Clinical and pathologic features of AEG samples used in this study.

SUPPLEMENTARY TABLE S2 DEGs of AEG bulk RNA-seq.

SUPPLEMENTARY TABLE S3 DEGs of AEG-like malignant cells in scRNA-seq.

SUPPLEMENTARY TABLE S4 GEP gene sets.

SUPPLEMENTARY TABLE S5 Primers used for real time-qPCR and ChIP-qPCR analysis.
