# Supplementary material for: Malignant epithelia cells-derived spermine induces APOE+ macrophages to suppress tumor immunity in adenocarcinoma of the esophagogastric junction
Source: Front Med (Lausanne). 2025 Sep 2;12:1636699. doi: 10.3389/fmed.2025.1636699 (PMC12436273; doi:10.3389/fmed.2025.1636699)
Supplement: Supplementary file 6 [file Data_Sheet_1.PDF]

Supplementary Figures

Figure S1

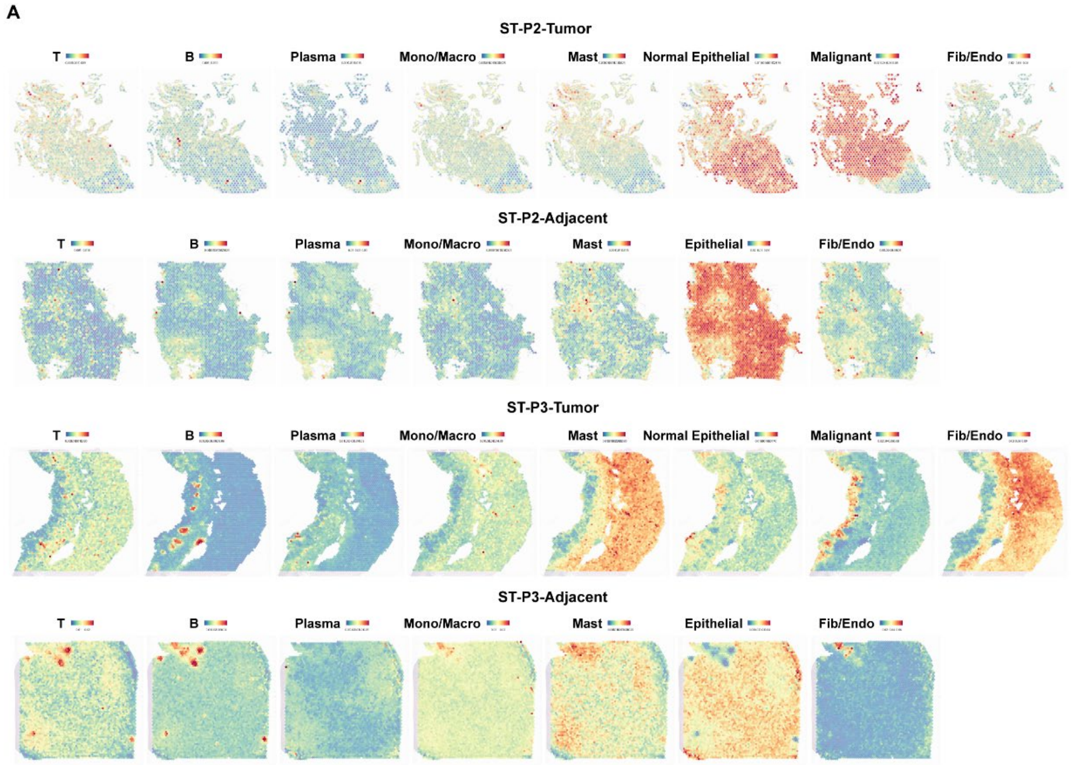

Figure S1 Major cell type proportions in ST data.

A. Spatial feature plots displaying the proportions of major cell types in each ST slides, proportion is indicated by color. Proportions of normal epithelial cells and malignant cells are distinctly displayed in tumor samples.

Figure S2

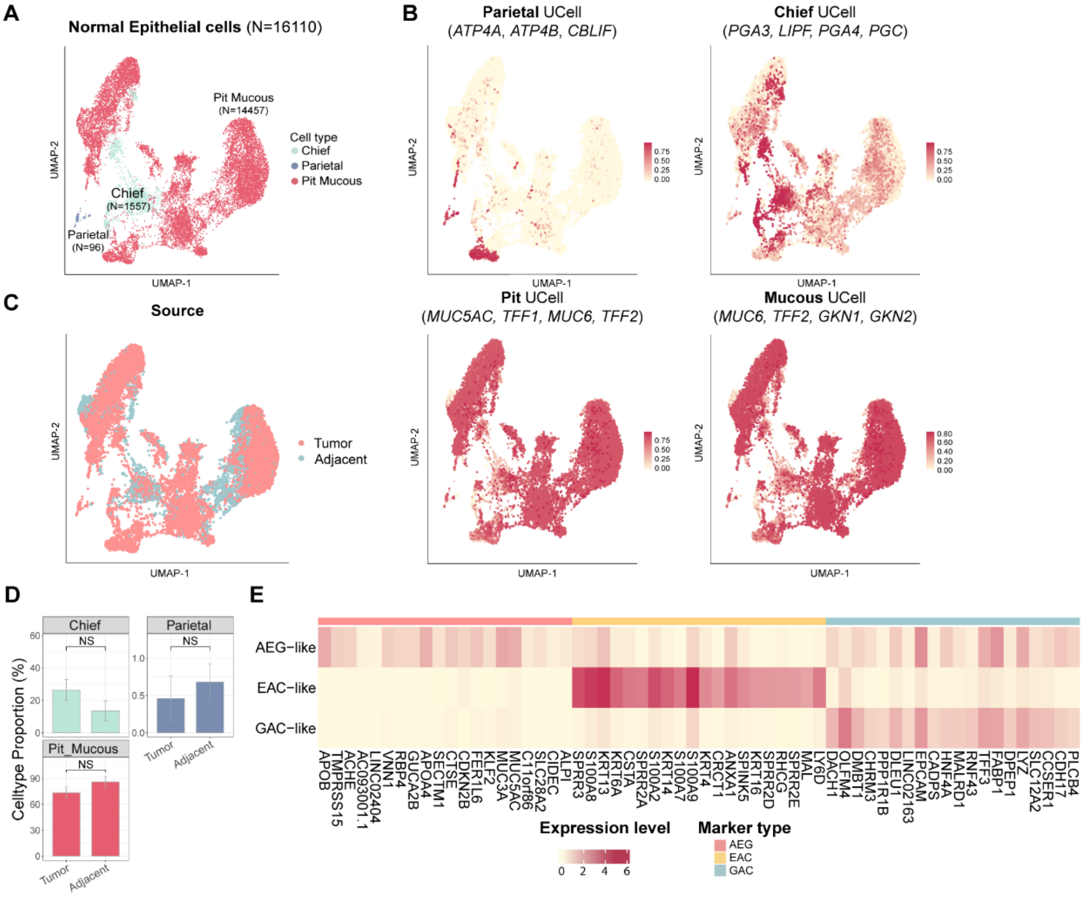

Figure S2 Characterization of normal epithelial major cell types.

A. UMAP of 16110 normal epithelial cells, colored by 3 major cell types.

B. UMAPs of UCell scores of marker genes for major cell types in normal epithelial cells. The major maker genes used in calculate UCell scores were labeled in the title below the major cell type.

C. UMAP showing sample sources of normal epithelial cells, colored by sample source type.

D. Boxplots showing the cell type proportions of major normal epithelial cell types in tumor and adjacent samples, colored by cell type (*P,* two-sided Wilcoxon test, NS: $P > 0.05$).

E. Heatmap showing tumor-specific marker gene expressions in AEG-like, EAC-like and GAC-like malignant cell subtypes.

Figure S3

A

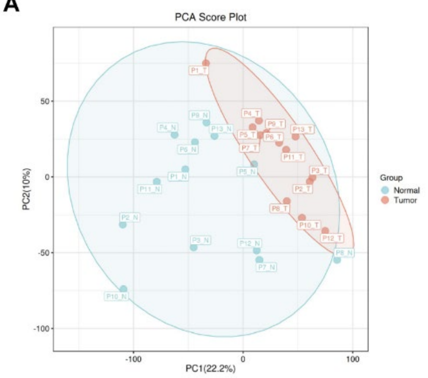

Figure S3 Characterization of AEG metabolic state.

A. PCA plot of 26 paired tumor and adjacent samples of LC-MS untargeted metabolomics of AEG, colored by sample source.

Figure S4

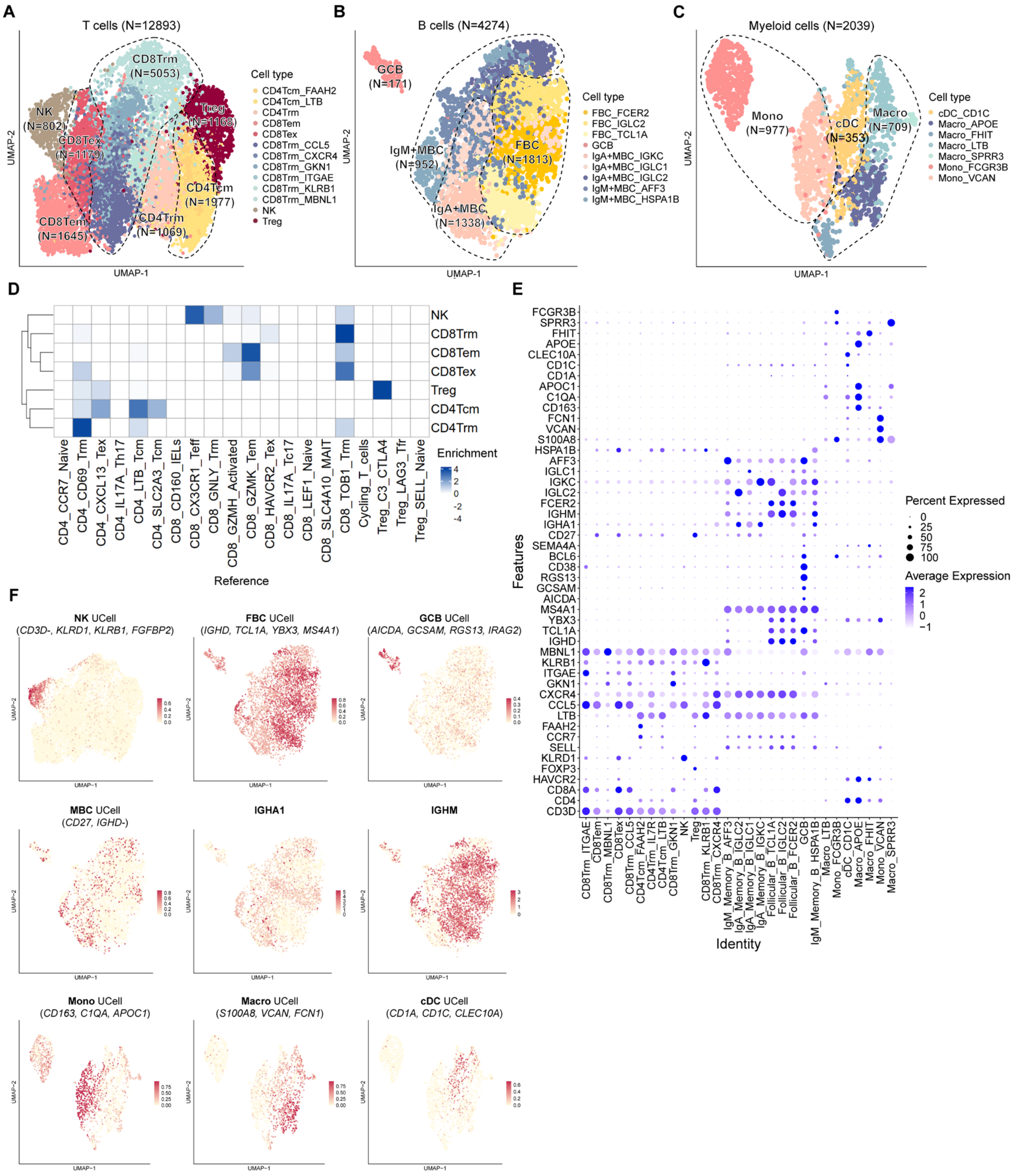

Figure S4 Characterization of AEG immune microenvironment.

A. UMAP of all 12893 T cells, colored by minor cell types. The major cell types are marked by dot lines.

B. UMAP of all 4274 B cells, colored by minor cell types. The major cell types are marked by dot lines.

C. UMAP of all 2039 myeloid cells, colored by minor cell types. The major cell types are marked by dot lines.

D. Heatmap showing the similarity between T minor cell types with reference T cell types. Columns are minor T cell types in reference scRNA-seq dataset, rows are T cell types in our scRNA-seq dataset.

E. Dot plot showing the expression levels of marker genes of T, B and myeloid cell

subtypes, expression level is indicated by color and percent of cells expressed target gene is indicated by dot size.

F. UMAPs showing the UCell scores or expression levels of marker genes of immune cell subtypes. The major maker genes used in calculate UCell scores were labeled in the title below the cell type.

Figure S5

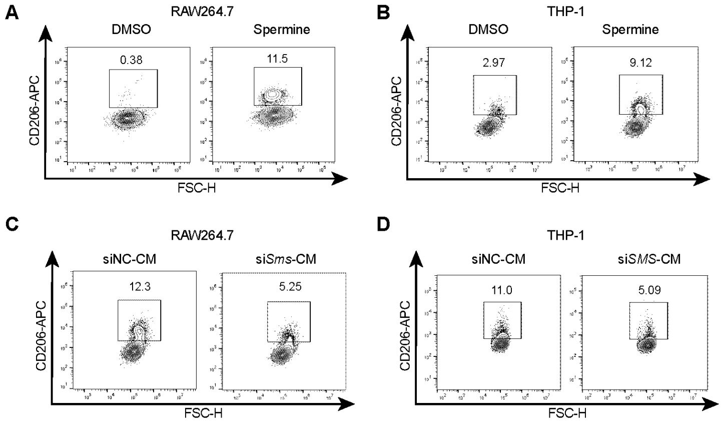

Figure S5 Spermine activates the STAT3/APOE axis in macrophage in vitro.

A. Flow cytometry gating strategy for CD206+ cells in RAW264.7 treated with DMSO or spermine (10uM) for 48h.

B. Flow cytometry gating strategy for CD206+ cells in THP-1 treated with DMSO or spermine (10uM) for 48h.

C. Flow cytometry gating strategy for CD206+ cells in RAW264.7 treated with CM of control or siSMS for 48h

D. Flow cytometry gating strategy for CD206+ cells in THP-1 treated with CM of control or siSMS for 48h.
