# Supplementary material for: Malignant epithelia cells-derived spermine induces APOE+ macrophages to suppress tumor immunity in adenocarcinoma of the esophagogastric junction
Source: Front Med (Lausanne). 2025 Sep 2;12:1636699. doi: 10.3389/fmed.2025.1636699 (PMC12436273; doi:10.3389/fmed.2025.1636699)
Supplement: Supplementary file 7 [file Presentation_1.PPTX]

## Slide 1
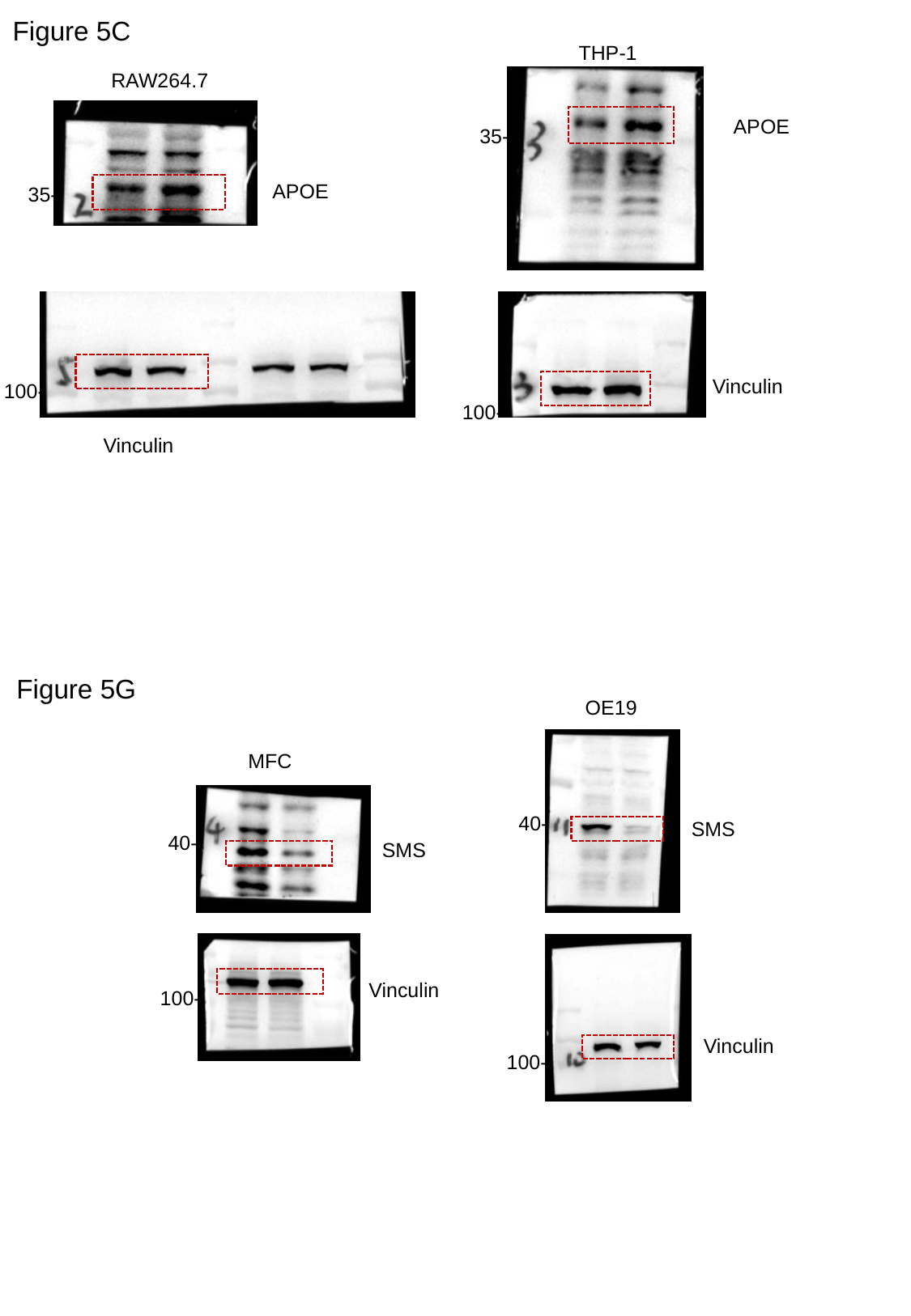

Figure 5C
THP-1
RAW264.7
APOE
35-
APOE
35-
Vinculin
100-
100-
Vinculin
Figure 5G
OE19
MFC
40-
SMS
40-
SMS
Vinculin
100-
Vinculin
100-

## Slide 2
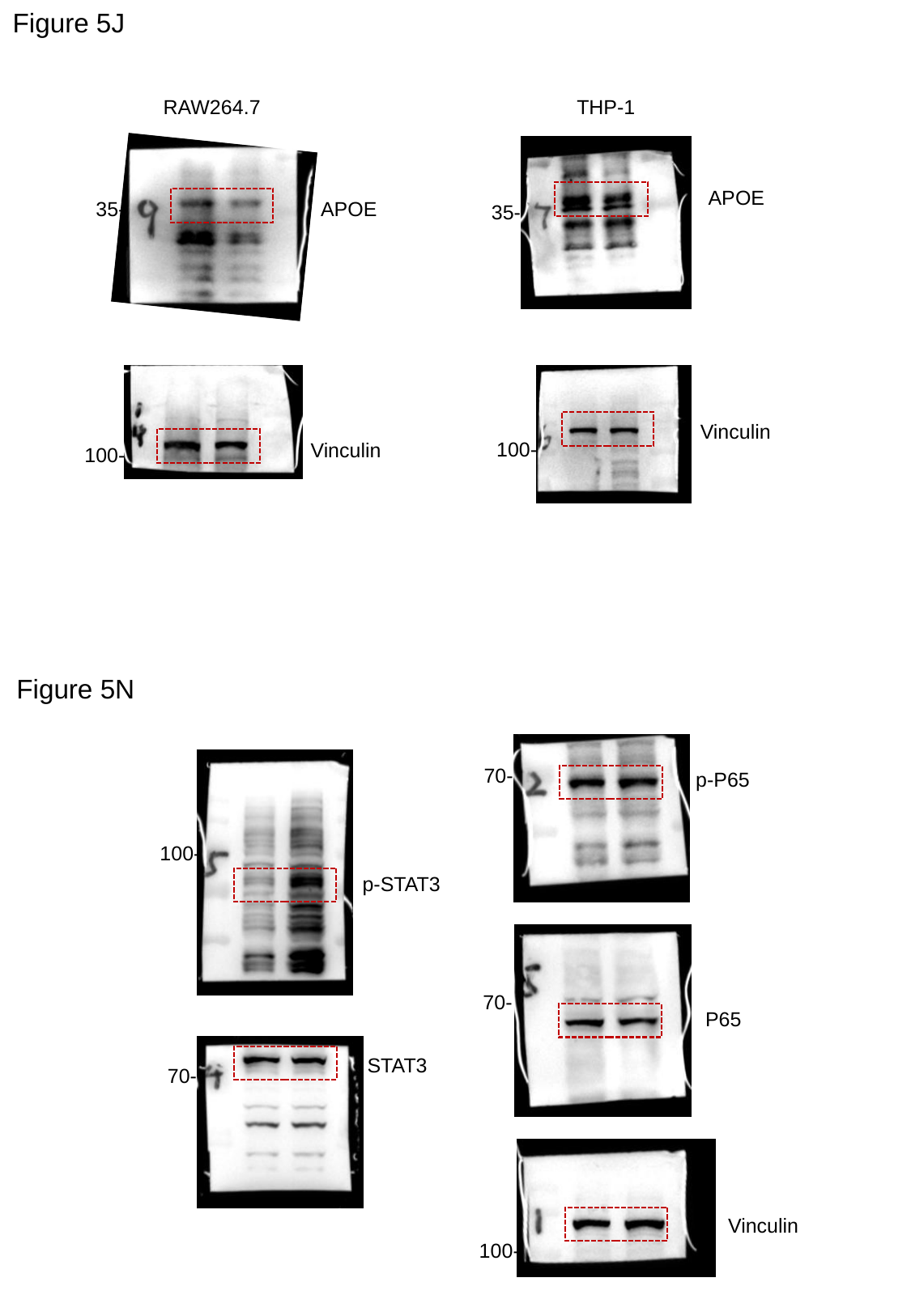

Figure 5J
THP-1
RAW264.7
APOE
APOE
35-
35-
Vinculin
100-
Vinculin
100-
Figure 5N
70-
p-P65
100-
p-STAT3
70-
P65
STAT3
70-
Vinculin
100-

## Slide 3
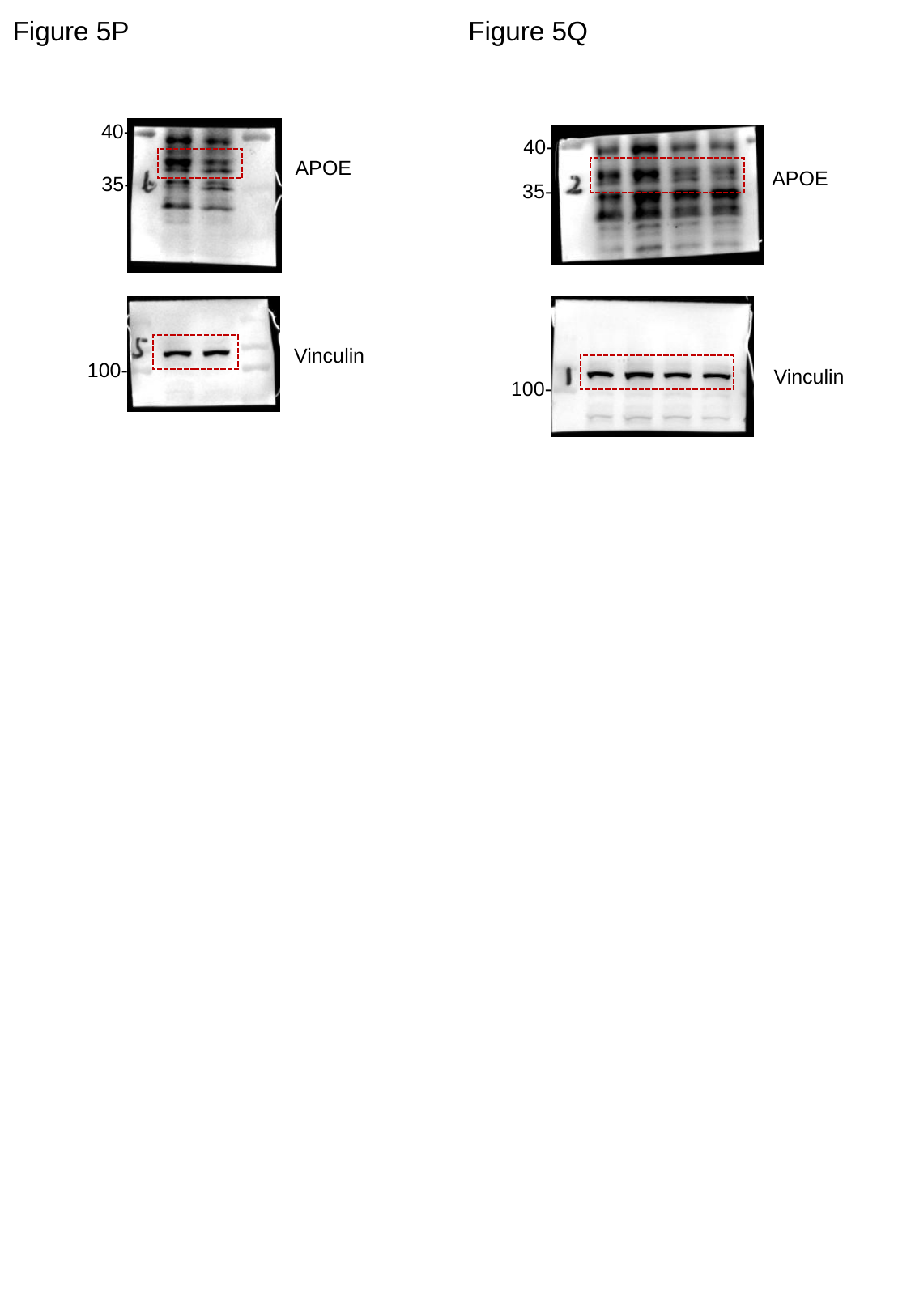

Figure 5P
Figure 5Q
40-
40-
APOE
APOE
35-
35-
Vinculin
100-
Vinculin
100-
